# Mapping RNA–RNA interactome and RNA structure *in vivo* by MARIO

**DOI:** 10.1038/ncomms12023

**Published:** 2016-06-24

**Authors:** Tri C. Nguyen, Xiaoyi Cao, Pengfei Yu, Shu Xiao, Jia Lu, Fernando H. Biase, Bharat Sridhar, Norman Huang, Kang Zhang, Sheng Zhong

**Affiliations:** 1 Department of Bioengineering, University of California, San Diego, Powell-Focht Bioengineering Hall 384, 9500 Gilman Drive, MC 0412, La Jolla, California 92093, USA; 2 Department of Ophthalmology, University of California, San Diego, La Jolla, California 92093, USA

## Abstract

The pervasive transcription of our genome presents a possibility of revealing new genomic functions by investigating RNA interactions. Current methods for mapping RNA–RNA interactions have to rely on an ‘anchor' protein or RNA and often require molecular perturbations. Here we present the MARIO (Mapping RNA interactome *in vivo*) technology to massively reveal RNA–RNA interactions from unperturbed cells. We mapped tens of thousands of endogenous RNA–RNA interactions from mouse embryonic stem cells and brain. We validated seven interactions by RNA antisense purification and one interaction using single-molecule RNA–FISH. The experimentally derived RNA interactome is a scale-free network, which is not expected from currently perceived promiscuity in RNA–RNA interactions. Base pairing is observed at the interacting regions between long RNAs, including transposon transcripts, suggesting a class of regulatory sequences acting in *trans*. In addition, MARIO data reveal thousands of intra-molecule interactions, providing *in vivo* data on high-order RNA structures.

## Abstract (web summary)

Current methods for mapping RNA-RNA interactions have to rely on an ‘anchor' protein or RNA. Here, the authors report the MARIO (Mapping RNA interactome *in vivo*) technology that can massively reveal RNA-RNA interactions and RNA structure from unperturbed cells.

## Graphical abstract

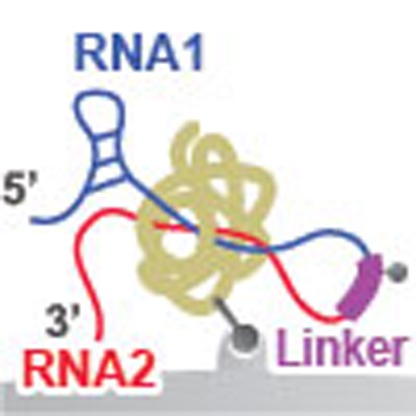

More than 97% of the human genome are non-coding DNA, of which >98% do not appear to be *cis*-regulatory sequences^1^,^2^,^3^,^4^. Thus, the functions of the vast majority of the human genome remains unknown. We noted that >85% of the human genome is transcribed into RNA^5^,^6^. Important functions of several classes of RNA were discovered by studying RNA–RNA interactions. These discoveries include that transfer RNA interacts with messenger RNA to translate genetic code^7^,^8^, and that microRNA (miRNA) interacts with mRNA to promote their degradation^9^,^10^,^11^, as well as interactions related to RNA splicing, editing and ribosomal RNA maturation. Are there other unknown RNA–RNA interactions with important functions? The technical challenge lies in the astronomical number of possible RNA pairs. A genome-wide technology for identifying RNA interactions *in vivo* is much needed.

Interactions between RNA molecules are often mediated by RNA-binding proteins^12^ such as ARGONAUTE proteins^13^, PUM2, QKI^14^ and small nucleolar RNA proteins^15^. However, it is difficult to directly observe RNA–RNA interactions facilitated by single proteins in normal cellular conditions. CLASH^16^,^17^ and hiCLIP^18^ use transformed cell lines that overexpress the facilitating protein. It is unclear to what extent that ectopic expression or genome-insertion-based cell transformation would influence RNA–RNA interactions. PAR-CLIP^14^ and HITS-CLIP^19^ assay RNAs attached to an RNA-binding protein, which do not directly assay RNA–RNA interactions. Most importantly, all the methods above trace the interactions ‘anchored' at a known protein or RNA. It is infeasible to map the entire RNA–RNA interactomes by extensions of these one-RNA-at-a-time or one-protein-at-a-time methods.

As the previous technologies relied on an ‘anchor' RNA or protein, the topology of the entire RNA–RNA interactome remains unknown. Inferring from the notion that regulatory RNAs ‘promiscuously' interact with 300–1,000 target RNAs^11^,^20^, one would probably guess that the RNA–RNA interactome has a flat topology, as opposed to a hierarchical topology^21^,^22^ that is shared by many other biological networks^21^,^22^.

The MARIO technology maps RNA–RNA interactions in a massive scale. MARIO can identify protein-assisted between-molecule and within-molecule RNA interactions. The MARIO identified RNA–RNA interactome is composed of tens of thousands of interactions, which involve mRNA, long intergenic noncoding RNA (lincRNA), small nucleolar RNA (snoRNA), small nuclear RNA, tRNA, miRNA, transposon RNA, pseudogene RNA, antisense RNA and novel transcripts. The MARIO identified RNA–RNA interactome is a scale-free network. Long non-coding RNA including lincRNA, transposon RNA and pseudogene RNA are observed to interact with mRNA. Sequence complementation is observed in interactions between transposon (LINE and LTR) RNA and mRNA, as well as in mRNA–mRNA, mRNA–peudogeneRNA, lincRNA–mRNA, miRNA–mRNA and LINE–miRNA interactions. MARIO data also provide spatial-proximity information related to RNA folding in three-dimentional space.

## Results

### The MARIO technology

**Figure 1:**
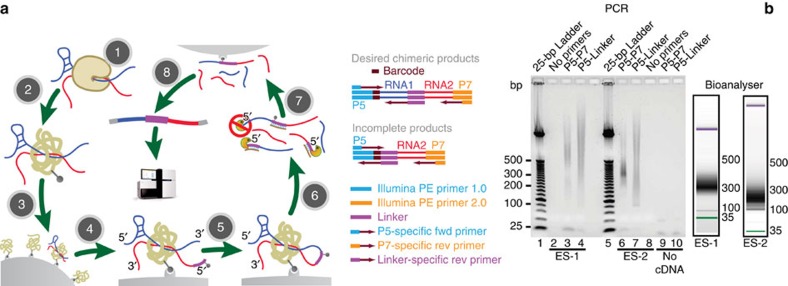
MARIO technology. (**a**) The major experimental steps are as follows: (1) cross-linking RNAs to proteins; (2) RNA fragmentation, protein denaturing and biotinylation; (3) immobilization of RNA-binding proteins at low density; (4) ligation of a biotinylated RNA linker; (5) proximity ligation under a dilute condition; (6) RNA purification and RT; (7) biotin pull-down; and (8) construction of sequencing library. (**b**) PCR validation of RNA1–Linker–RNA2 chimeras, which were expected to be above 91 bp from the P5 sequencing primer to the linker (purple) and above 200 bp from P5 to P7 sequencing primers. The failure to include RNA1 would create 91 bp products from P5 to the linker. The failure to include RNA2 would create similar-sized products from P5 to the linker and from P5 to P7. The PCR primers are marked on top of each lane. The size distribution of the sequencing libraries was also assessed by Bioanalyzer.

We developed the MARIO technology to detect RNA–RNA interactions facilitated by any single protein *in vivo*. In this procedure, RNA molecules are cross-linked with their bound proteins and then ligated to a biotinylated RNA linker such that proximal RNA molecules co-bound by the same protein form a chimeric RNA in the form of RNA1–Linker–RNA2. These linker-containing chimeric RNAs are isolated using streptavidin-coated magnetic beads and subjected to paired-end sequencing (Fig. 1a and Supplementary Fig. 1). Thus, each non-redundant paired-end read reflects a molecular interaction.

We carried out two independent MARIO assays on mouse embryonic stem (ES) cells with minor technical differences (Supplementary Table 1 and Supplementary Figs 2–5), which we designated as ES-1 and ES-2. A library for indirect RNA interactions was produced using two cross-linking agents (formaldehyde and EthylGlycol bis (SuccinimidylSuccinate))^23^,^24^,^25^, which ‘effectively captures RNAs linked indirectly through multiple protein intermediates'^26^ (ES indirect). Two other unique libraries were produced from mouse embryonic fibroblasts (MEFs) and mouse brain, offering two additional data sets for bioinformatic quality assessment (Supplementary Fig. 6). We confirmed that each library contained RNA constructs of the desired form (RNA1–Linker–RNA2) and lengths (Fig. 1b). We sequenced each library to yield, on average, 47.3 million paired-end reads, among which ∼15.1 million non-redundant paired-end reads represented the desired chimeric form (Supplementary Fig. 7). In addition, we carried out three control experiments. The first and the second control experiments excluded the cross-linking step (non-cross-linking control) and the protein biotinylation step (non-biotinylation control), respectively (Supplementary Note 1). The third control experiment used *Drosophila* S2 cells and mouse ES cells to test the extent of random ligation of RNAs (cross-species control). After cross-linking and cell lysis, the lysates from the two cell lines were immediately mixed before any subsequent steps. The mixture was subjected to the rest of the experimental procedure and resulted in a sequenced library (Fly-Mm). The proportion of RNA pairs mapped to two species is in the range of 2.5–6.8%, depending on whether the *Drosophila* genome and the mouse genome were assembled into a pan genome^16^,^27^ before mapping (Supplementary Note 1). We chose the more conservative estimate (derived from mapping to the pan genome) that 6.8% of the ligation products were generated from random ligations. This estimate is comparable to that (7.0%) derived from *in silico* simulations (Supplementary Note 2).

A suite of bioinformatics tools was created (MARIO tools) to analyse and visualize MARIO data. MARIO tools automated the analysis steps, including removing PCR duplicates, splitting multiplexed samples, identifying the linker sequence, splitting junction reads, calling interacting RNAs, performing statistical assessments, categorizing RNA interaction types, calling interacting sites and analysing RNA structure (http://mariotools.ucsd.edu). It also provides visualization tools for both the RNA–RNA interactome and the proximal sites within each RNA (Supplementary Fig. 8).

### RNA–RNA interactome in ES cells

We compared the five MARIO libraries. ES-1 and ES-2 were most similar as judged by correlations of FPKMs (fragments per kilobase of transcript per million mapped reads; separately calculated for the read fragments on the left and the right sides of the linker), followed by ES indirect, MEF, and then brain tissue (Supplementary Fig. 6). The interacting RNA pairs identified from ES-1 and those from ES-2 exhibited strong overlaps (*P*-value<10^−35^, permutation test) (Supplementary Table 2). The interactions identified in MEF did not exhibit significant overlaps with those in either of the ES samples (*P*-value for each overlap=1, permutation tests). For example, an interaction between the 3′ untranslated region of *Trim25* mRNA and *Snora1* snoRNA was supported by multiple paired-end reads in ES-1 and ES-2 samples, but was not detected in MEF (Supplementary Fig. 7). We did not expect many interactions identified from ES-1 and ES-2 to show up in ES indirect data, because a cross-linked protein complex can bury an RNA molecule, limiting the RNA's accessibility to RNA ligase, which is required to form the chimeric RNA product. Among the snoRNAs identified as having interactions with mRNAs in our data sets, 172 of them, including *Snora1*, were detected both as enzymatically processed small RNAs^28^ (red lane, Supplementary Figs 7 and 9–11) and in ARGONAUTE HITS-CLIP data (Supplementary Figs 9–11). This supports the proposition that transcripts from snoRNA genes could be enzymatically processed into miRNA-like small RNAs and interact with mRNAs in RISC complex^29^,^30^ (Supplementary Note 3).

**Figure 2:**
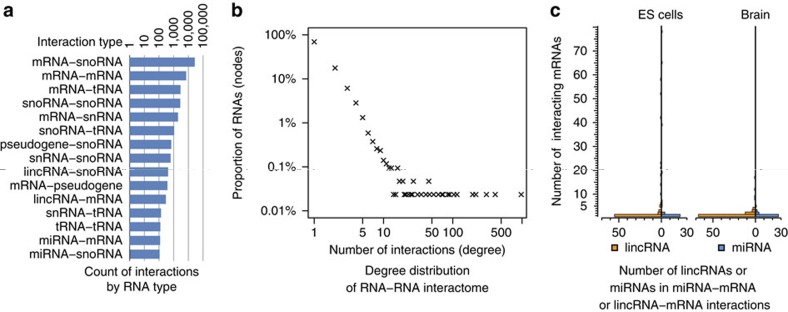
MARIO-identified RNA–RNA interactions. (**a**) Distribution of MARIO-identified RNA–RNA interactions among different types of RNAs in ES cells. rRNAs were experimentally and computationally removed from the analysis. (**b**) Degree distribution of the ES cell RNA–RNA interactome composed of mRNA, lincRNA, miRNA, pseudogene transcripts and antisense transcripts. The number of RNAs (as a proportion of all RNAs in the network, *y* axis) is inversely correlated with the number of interactions they participated (degree, *x* axis) in log scale, characteristic of scale-free networks. (**c**) The numbers of lincRNAs and miRNAs (*x* axis) categorized by the number of their interacting mRNAs (*y* axis).

The ES-1 and ES-2 libraries were merged to infer the RNA–RNA interactome in ES cells. This dataset included 4.54 million non-duplicated paired-end reads that were unambiguously split into two RNA fragments with both fragments uniquely mapping to the genome (mm9). We identified tens of thousands of inter-RNA interactions (false discovery rate <0.05, Fisher's exact test with Benjamin–Hochberg correction) (Supplementary Fig. 12). As expected, the RNA expression level (FPKM) is weakly correlated with the number of MARIO reads on each RNA, but FPKM is not correlated with the statistical significance (false discovery rate) of the interactions (Supplementary Fig. 12C,D). mRNA–snoRNA interactions were the most abundant type, although thousands of mRNA–mRNA and hundreds of lincRNA–mRNA, pseudogeneRNA–mRNA and miRNA–mRNA interactions were also detected (Fig. 2a). Our simulation suggested ∼66% sensitivity and ∼93% specificity for the entire experimental and analysis procedure (Supplementary Note 2).

### Validation of selected interactions

**Figure 3:**
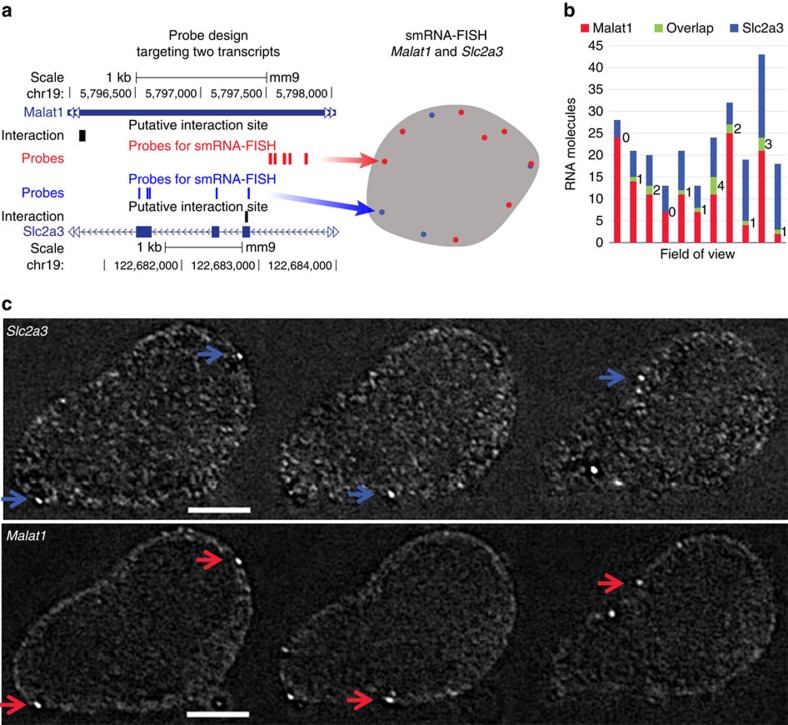
Testing co-localization of *Malat1* lincRNA and *Slc2a3* mRNA with smRNA-FISH. (**a**) Positions of hybridization probes labelled with 60- (red) and 525-nm (blue) qDots. (**b**) Quantification of *Malat1* and *Slc2a3* RNA molecules. Green: number of co-localized molecules. (**c**) Representative images of two-colour smRNA-FISH showing four co-localized molecules (arrows). Scale bar, 10 μm.

We used two methods to validate selected interactions in the MARIO identified interactome. These two methods were selected because they do not perturb the cells or change RNA expression levels. First, we examined co-localization between *Malat1* lincRNA and *Slc2a3* mRNA *in vivo* by two-colour single-molecule RNA fluorescence *in situ* hybridization (smRNA–FISH)^31^. Quantum dots (qDots) were used instead of organic dyes for increased fluorescence signal intensities and narrower ranges of emission wavelengths^32^. When designed to target the same transcript, the qDot based (five 25–30-nt probes) and the organic dye-based (forty-three 20-nt hybridization probes) smRNA–FISH identified the same *Actb* mRNAs in the cytoplasm (Supplementary Figs 13 and 14). We designed probes for *Malat1* and *Slc2a3* (Fig. 3a) and labelled them with 605 and 525 nm qDots, respectively, for an unequivocal distinction of signal from each qDot (Supplementary Fig. 15). We note that qDots cannot penetrate into the nuclei^33^ unless specific delivery methods are used^34^, and in mouse ES cells approximately a quarter of *Malat1* RNA is in the cytoplasm^35^. Cytoplasmic *Malat1* and *Slc2a3* RNAs were detected in 27 ES cells, with an average of 7.6 and 4.5 copies per cell, respectively (Supplementary Fig. 16). We called co-localization of two RNAs with a threshold on the distance between the estimated centres of two FISH spots, which corresponds to a physical distance <0.2 μm (ref. 36). Sixteen pairs of co-localized *Malat1* and *Slc2a3* RNA molecules were detected from a total of 80 copies of *Malat1* and 50 copies of *Slc2a3* (Fig. 3b,c). In the first control experiment, we detected 1 co-localization of *Malat1* and *Actb* RNAs from 124 copies of *Malat1* and 34 copies of *Actb*. In the second control experiment, we detected 1 co-localization of *Slc2a3* and *Actb* RNAs from 545 copies of *Slc2a3* and 298 copies of *Actb* (odds ratio between experiment (*Malat1*-*Slc2a3*) and controls (*Malat1*-*Actb* and *Slc2a3-Actb*)=446, *P*-value <10^−20^, *χ*^2^-test).

To test interactions at a larger scale, we carried out RNA interactome analysis and sequencing (RIA-seq)^37^. We chose RIA-seq, because it does not require genetic perturbation. First, we did Malat1 RIA-seq and Actb RIA-seq (control) to test the interactions involving Malat1 (Supplementary Note 4). Malat1 RNA itself exhibited a 5.81-fold increase in Malat1 RIA-seq over Actb RIA-seq, confirming the validity of the RIA purification. Malat1-interacting RNAs reported by MARIO showed 14.6 (0610007P14Rik), 4.53 (Slc2a3), 3.38 (Eif4a2) and 2.39 (Tfrc)-fold increase in Malat1 RIA-seq over Actb RIA-seq (*P*-value<0.0003, *χ*^2^-test). This suggests a strong overlap of Malat1 targets in MARIO and Malat1 RIA-seq. Next, we asked whether Tfrc RIA could reversely identify Malat1 by Tfrc RIA-seq (Supplementary Note 4). The Tfrc RNA itself showed 2.87-fold of increase in Tfrc RIA-seq compared with Actb RIA-seq. Malat1 exhibited 3.84-fold increase (*P*-value<2.2 × 10^−16^, derived from testing the null hypothesis fold change=1), suggesting that antisense purification of Tfrc could reversely pull down Malat1. In addition, three out of four other Tfrc-interacting RNAs identified by MARIO exhibited 1.4- to 13.6-fold increases (*P*-value<0.00002, *χ*^2^-test). Taken together, seven additional MARIO-identified interactions were validated by RIA-seq.

### MARIO-derived RNA–RNA interactomes are scale free

The experimentally derived RNA interactome offered an opportunity to test a fundamental physical property. Most known biological networks are hierarchical networks^21^,^22^, where the number of nodes is reversely correlated with the number of interactions (edges) they participate. This reverse correlation takes a linear form in log scale, which is called the power law, also referred to as the scale-free property^38^. However, RNA–RNA interactions have been reported as ‘surprisingly promiscuous'^20^. It was suggested that each miRNA interacts with 300–1,000 mRNAs in one cell type^19^ and a similar picture was proposed for lincRNAs^39^. Extrapolating from these information, one would not expect the RNA interactome to be scale free. For example, an artificial network with 100 miRNAs and each miRNA randomly connected to 300–1,000 mRNAs is not a hierarchical network (Supplementary Fig. 17A).

The MARIO-derived RNA–RNA interactome is scale free (Fig. 2b and Supplementary Fig. 17B). In other words, the quantity of RNAs with a given number of interaction partners (the number of RNAs at the same level of promiscuity) decreases exponentially as that number of interaction partners (promiscuity) increases. In theory, the scale-free property of the experimentally derived network should not be affected by the bioinformatic threshold used for calling the interactions. This is because uniformly adding or withdrawing edges at random should not affect the log linearity in the network's degree distribution. We tested this empirically. Indeed, changing the threshold of calling interactions did not affect the log linearity between the number of RNAs and their number of interaction partners (Supplementary Fig. 17D). Furthermore, the scale-free property of the observed RNA interactome does not change if we remove all rRNA and snoRNA from the network (Fig. 2b). In addition, the RNA interactome derived from mouse brain is scale free (Supplementary Fig. 17C), suggesting this global property is not cell-type specific. In each cell type, there are exponentially more miRNAs and lincRNAs that had relatively specific (fewer) mRNAs than those with greater number of targets (Fig. 2c). In summary, regardless of the thresholds and cell types, MARIO-derived RNA interactomes were hierarchical networks. Therefore, MARIO-derived RNA interactome shares the most essential network property (scale free) as other types of known biological networks. We speculate that this fundamental topological property has not been reported, because previous methods requiring ectopic expression or genetic perturbation may affect the network topology by altering the concentrations of critical molecules.

### Frequently used RNA segments for interactions

**Figure 4:**
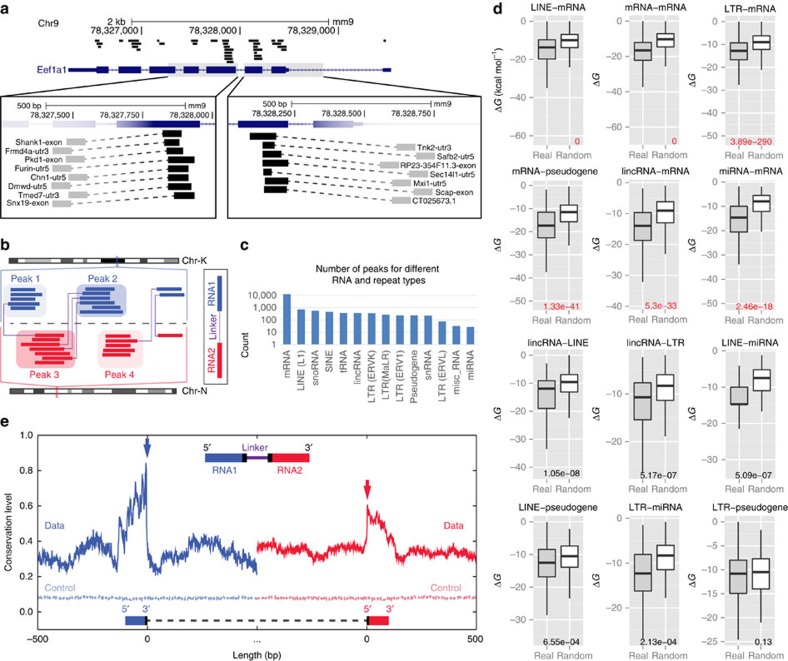
RNA interaction sites. (**a**) Multiple MARIO reads, representative of different interactions (dashed lines), overlapped on specific regions of the *Eef1a1* gene. (**b**) Finding interaction sites by the ‘peaks' of overlapping reads. (**c**) Distribution of interaction sites in different types of RNA genes and transposons. (**d**) The distribution of binding energies (Δ*G*, kcal mol^−1^) between the interaction sites of two RNAs (grey) and between randomly shuffled bases (white). *P*-values from Wilcoxon rank test are marked at the bottom of each panel. (**e**) Conservation levels, measured by average PhyloP scores peaked at the junction (black bar, position 0 on the *x* axis) of the ligated RNA fragments. Control: conservation levels of randomly selected genomic regions.

A number of the interacting RNAs exhibited overlapping MARIO reads (Fig. 4a), suggesting interactions were often concentrated at specific segments of an RNA. ‘Peaks' of overlapping read fragments were identified and termed ‘interaction sites' (Fig. 4b). Interaction sites appeared not only on miRNAs (the entire mature miRNA), mRNAs and lincRNAs, but also on pseudogene and transposon RNAs (Fig. 4c). Over 2,000 interaction sites were harboured in L1, SINE, ERVK, MaLR and ERV1 transposon RNAs (Supplementary Table 3), indicative of their frequent interactions with other RNAs^40^,^41^. In addition, pseudouridines^42^ were enriched in the mRNA interactions sites of snoRNA–mRNA interactions, corroborating the idea that some RNA segments were favoured in certain types of RNA interactions (Supplementary Note 5).

### Sequence complementation on RNA interaction regions

We asked whether base complementation is used by different types of RNA–RNA interactions. We estimated the hybridization energy of a pair of interacting RNAs by the average hybridization energy of all pairs of ligated fragments (RNA1 and RNA2)^43^ that were mapped to this RNA pair, and compared it with the hybridization energy of control fragment pairs generated by random shuffling of the bases. Complementary bases were preferred in nearly all types of RNA–RNA interactions and were most pronounced in transposonRNA–mRNA, mRNA–mRNA, pseudogeneRNA–mRNA, lincRNA–mRNA and miRNA–mRNA interactions (*P*-values<2.4^−18^, test by random shuffling), but was not observed in LTR–pseudogeneRNA interactions (Fig. 4d and Supplementary Fig. 18). This data led us to speculate that base pairing facilitates sequence-specific posttranscriptional regulation in long RNAs.

### Evolutionary conservation of interaction sites

If these RNA–RNA interactions are sequence specific^44^, the RNA interaction sites should be under selective pressure. We found that the interspecies conservation levels^45^ are strongly increased at the interaction sites and the peak of conservation precisely pinpointed the junction of the two RNA fragments (Fig. 4e). When interacting with lincRNAs, pseudogene RNAs, transposon RNAs or other mRNAs, the interaction sites on mRNAs were more conserved than the rest of the transcripts (Supplementary Fig. 19). The interactions sites on lincRNAs and pseudogene RNAs exhibited increased conservation in lincRNA–mRNA, pseudogeneRNA–mRNA and pseudogeneRNA–transposonRNA interactions (Supplementary Fig. 19). The increased conservation at interaction sites was not due to exon-intron boundaries (Supplementary Fig. 20). Taken together, base complementarity is widespread in the interactions of long RNAs. The complementary regions are evolutionarily conserved.

### Three-dimensional RNA structure

**Figure 5:**
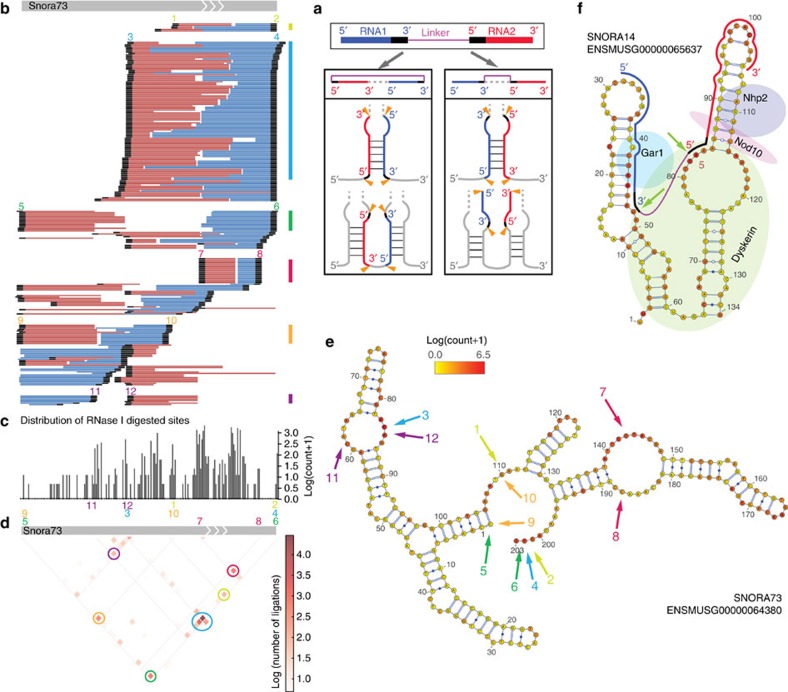
RNA structure. (**a**) Schematic depiction of resolving the proximal sites of an RNA. Orange arrow: RNase I cutting site. (**b**) The ‘cut and ligated' products mapped to *Snora73*. Black regions: ligation junctions. Vertical colour bar: a cluster of read pairs supporting a pair of proximity sites. (**c**) Density of RNase I cuts. (**d**) Heatmap of the ligation frequencies between any two positions of the RNA. Each coloured circle corresponds to a vertical colour bar in **a** and represents a pair of proximal sites. (**e**) Footprint of single-stranded regions (red in colour scale) and inferred proximal sites (arrows of the same colour) on the accepted secondary structure. (**f**) A pair of inferred proximal sites, which were not supported by sequenced-based secondary structure, are physically close *in vivo*, supposedly due to protein-assisted RNA folding.

Although we originally designed MARIO for mapping inter-molecule interactions, we also found that MARIO revealed RNA secondary and tertiary structures. All the analyses above were based on intermolecular reads. By looking at intramolecular reads, we learned two characteristics of RNA structure. First, the footprint of single-stranded regions of an RNA were identified by the density of RNase I digestion sites (RNase I digestion was applied before ligation, see Step 2 in Fig. 1a and Supplementary Fig. 21). Second, the spatially proximal sites of each RNA were captured by proximity ligation (Step 5 in Fig. 1a). Over 60,000 linker-containing read pairs were mapped to individual genes and thus were determined as intramolecule cutting and ligation (Supplementary Fig. 22A). Each cut-and-ligated sequence can be unambiguously assigned to one of two structural classes by comparing the orientations of RNA1 and RNA2 in the sequencing read with their orientations in the genome (Fig. 5a). These reads provided spatial proximity information for 2,374 RNAs, including those from 1,696 known genes and 678 novel genes. For example, 277 cut-and-ligated sequences were produced from *Snora73* transcripts (Fig. 5b). The density of RNase I digestion sites (Fig. 5c) was strongly predictive of the single-stranded regions of the RNA (heatmap; Fig. 5e). Six pairs of proximal sites were detected (circles; Fig. 5d). Each pair was supported by three or more cut-and-ligated sequences with overlapping ligation positions (black spots; Fig. 5b). Five out of the six proximal site pairs were physically close in the generally accepted secondary structure (arrows of the same colour; Fig. 5e). On *Snora14*, a pair of inferred proximal sites appeared distant, according to sequenced inferred secondary structure (green arrows; Supplementary Fig. 23). However, ribonucleoprotein DYSKERIN bent *Snora14* transcript *in vivo*^46^,^47^, making the two pseudouridylation loops close to each other, as predicted by the cut-and-ligated sequence (green arrows; Fig. 5f). Structural information can even be derived on novel transcripts and some parts of mRNAs (Supplementary Figs 24 and 25). To date, resolving the spatially proximal bases of any individual RNA in three-dimensional space remains a grand challenge. MARIO provides intra-molecule spatial proximity information for the thousands of RNAs. In addition, the single-strand footprints of every RNA are mapped at the same time. Thus, MARIO largely expanded our capacity to examine RNA structures.

## Discussion

The MARIO method offers several advantages for mapping RNA–RNA interactions. First, MARIO directly analyses the endogenous cellular features without introducing any exogenous nucleotides^14^,^47^,^48^ or protein-coding genes^16^ before cross-linking. This eliminates the uncertainty of reporting spurious interactions produced by changing the RNA or protein expression levels. Moreover, it makes MARIO well-suited for assaying tissue samples. Second, the introduction of a selectable linker enables an unbiased selection of interacting RNAs, making it possible to globally map an RNA–RNA interactome. This method circumvents the requirement for a protein-specific antibody or the need to express a tagged protein. It also removes the limit of working with one RNA-binding protein at a time. Third, this method only captures the RNA molecules co-bound with a single protein molecule, avoiding capture of RNA molecules that are independently bound to different copies of a protein^14^,^19^, which would potentially lead to reporting spurious interactions. Fourth, false positives that result from RNAs ligating randomly to other nearby RNAs are minimized by performing the RNA ligation step on streptavidin beads in extremely dilute conditions. Fifth, the RNA linker provides a clear boundary delineating the position of ligation site in the sequencing reads, thus avoiding ambiguities in mapping the ligated chimeric RNA. Sixth, potential PCR amplification biases are removed by attaching a random four- or six-nucleotide barcode to each chimeric RNA before PCR amplification and subsequently counting completely overlapping sequencing reads with identical barcodes only once^19^,^49^,^50^,^51^,^52^. MARIO should facilitate future investigations of RNA functions and regulatory roles.

## Methods

### Cell culture

Undifferentiated mouse E14 ES cells (gift from Huck-Hui Ng) were cultured under feeder-free conditions. ES cells were seeded on gelatin-coated dishes and were cultured in DMEM medium (GIBCO) supplemented with 15% fetal bovine serum (FBS; Gemini Gemcell), 0.055 mM 2-mercaptoethanol (Sigma), 2 mM Glutamax (GIBCO), 0.1 mM MEM non-essential amino acid (GIBCO), 5,000 U ml^−1^ penicillin/streptomycin (GIBCO) and 1,000 U ml^−1^ of LIF (Millipore). The cells were maintained in an incubator at 37 °C and 5% CO_2_.

MEFs (C57BL/5, GlobalStem) were cultivated in 15-cm dishes in DMEM (GIBCO) supplemented with 15% FBS (Gemini Gemcell), 0.055 mM 2-mercaptoethanol (Sigma), 2 mM Glutamax (GIBCO), 0.1 mM MEM non-essential amino acid (GIBCO), 5,000 U ml^−1^ penicillin/streptomycin (GIBCO). MEFs were also maintained in an incubator at 37 °C and 5% CO_2_.

*Drosophila* S2 cells (Invitrogen) were maintained in 15-cm plates in Schneider's *Drosophila* Medium (GIBCO) supplemented with 10% heat-inactivated FBS (Gemini Gemcell) and 5 ml 1:100 penicillin/streptomycin (GIBCO) in an incubator at 28 °C without CO_2_.

### Tissue dissection and preparation

Mice handling was approved by the Institutional Animal Care and Use Committee of the University of California, San Diego. Adult female (C57BL/6J background) was killed by cervical dislocation and the whole brain was immediately collected, rinsed with ice-cold PBS three times and snap frozen. Frozen, whole mouse brain tissue was ground into fine powder in liquid nitrogen using a mortar and pestle. The tissue powder was quickly transferred into a Petri dish on a bed of dry ice and irradiated on dry ice three times at 400 mJ cm^−2^ in an ultraviolet cross-linker (254 nm) with gentle swirling between each irradiation. Cross-linked, powdered tissue was immediately lysed and subjected to MARIO procedure as described.

### Overview of the MARIO method

MARIO was designed to: (i) capture interacting RNAs *in vivo* in an unbiased manner without genetically or transiently introducing exogenous molecules; (ii) allow stringent removal of non-physiologic associations that form after cell lysis^53^; (iii) select the proximity-ligated chimeric RNAs; and (iv) allow unambiguous bioinformatic identification of interacting RNAs. We achieved these objectives by: (i) cross-linking and immobilization of all RNA–protein complexes in streptavidin beads and removal of nonspecific binding by denaturing conditions; (ii) attaching a biotin-tagged RNA linker to facilitate selective enrichment of chimeric RNA constructs; (iii) using the linker sequence to unambiguously split the interacting RNAs from a sequencing read pair.

#### Cross-linking RNAs to proteins

Ultraviolet irradiation was used to form covalent bonds between photoreactive nucleotide bases and amino acids. Ultraviolet irradiation generates highly reactive, short-lived states of the nucleotide bases within the RNA, inducing covalent bond formation only with amino acids at their contact points without additional elements that might cause conformational perturbation^54^. Ultraviolet irradiation at 254 nm does not promote protein–protein cross-linking due to the different wavelengths absorbed by amino acids. Specifically, cells were washed twice in ice-cold PBS. E14 and MEF cells were irradiated once, whereas *Drosophila* S2 were irradiated three times with UV-C (254 nm) at 400 mJ cm^−2^ in ice-cold PBS on ice. Cells were harvested by scraping and pelleted by centrifugation at 1,000 *g* for 5 min at 4 °C. Cell pellets were snap-frozen in liquid nitrogen and stored at −80 °C.

We generated a MARIO library (ES indirect) in which we cross-linked protein–protein complexes as well. This was to capture the RNA that were brought together by protein interactions. We applied an *in vivo* dual cross-linking method with previously validated parameters^55^,^56^,^57^. Briefly, cells were first rinsed with room-temperature PBS and treated with 1.5 mM EthylGlycol bis (SuccinimidylSuccinate) (Pierce Protein Research Products, Rockford, Illinois) freshly prepared in PBS for 45 min at room temperature on a shaker. Cells were further treated with formaldehyde (Pierce Protein Research Products) to a final concentration of 1% and incubated for 20 min at room temperature with rocking. Glycine was added to a final concentration of 250 mM and incubated for 10 min at room temperature, to quench the cross-linking reaction. Cells were then washed once with PBS at room temperature, scraped off, pelleted at 1,000 *g* for 5 min at 4 °C, snap-frozen in liquid nitrogen and stored at −80 °C.

#### Cell lysis, RNA fragmentation, and protein biotinylation

Approximately 6 × 10^8^ cross-linked cells stored at −80 °C were thawed on ice and resuspended in ∼3 volumes of lysis buffer (50 mM Tris-HCl pH 7.5, 100 mM NaCl, 0.1% SDS, 1% IGEPAL CA-630, 0.5% sodium deoxycholate, 1 mM EDTA supplemented with 1:20 volume of EDTA-free complete protease inhibitor cocktail (Roche)). Lysis was performed on ice for 20 min. Cell debris and insoluble chromatin were removed by centrifugation at 20,000 *g* for 10 min at 4 °C. The supernatant was collected and treated with TURBO DNase (Invitrogen) at concentration of 10 μl TURBO DNase per ml lysate for 20 min at 37 °C. RNAs were digested into ∼1,000- to 2,000-nt (ES-1) or ∼1,000-nt (ES-2) fragments by adding 10 μl of 1:100 diluted RNase I (NEB) per ml of lysate and incubating at 37 °C for 3 min. Following RNase I treatment, the lysate was immediately transferred to ice for at least 5 min. Both RNase I and sonication-based fragmentation leave 5′-OH and 3′-P ends, incompatible with RNA ligation, which suppress undesirable RNA ligations. To stop DNase digestion, we added EDTA (Ambion) to a 25-mM final concentration and incubated the mixture at 4 °C for 15 min with rotation. The fragmented dual cross-linked (ES indirect) lysate was prepared as follows: after the lysis on ice for 20 min, the suspension was directly subjected to fragmentation by sonication (Covaris E220) under the following settings: 20 min with 5% duty cycle, 140 Watts peak incident power and 200 cycles per burst at 4 °C.

For cross-species experiment (Fly-Mm), ∼3 × 10^8^ E14 mES cells and 3 × 10^8^ *Drosophila* S2 cells were lysed separately and then mixed before protein biotinylation.

To dissociate loosely bound proteins, 500 mM NaCl final concentration was added and the solution was incubated at 4 °C for 10 min with rotation. To further dissociate protein complexes and non-cross-linked RNAs, and halt the activities of RNase I, we added SDS to a 0.3% final concentration and incubated the mixture with shaking at 750 r.p.m. for 15 min at 65 °C. After letting the solution mixture cool down to room temperature, we biotinylated the cysteine residues by adding to the lysate 1:5 volume of 25 mM (13.56 mg ml^−1^) EZlink Iodoacetyl-PEG2-Biotin (IPB) (Pierce Protein Research Products) and rotating the mixture in the dark for 90 min at room temperature. We quenched the biotinylation reaction by adding dithiothreitol (DTT) to a 5-mM concentration and incubating at room temperature for 15 min. To neutralize SDS, Triton X-100 (Sigma) was added to a 2% final concentration and incubated at 37 °C for 15 min. The lysate sample was dialysed in a 20-kDa cutoff Slide-A-Lyzer Dialysis Cassette (Pierce Protein Research Products) at room temperature in 2 l of dialysis buffer (20 mM Tris-HCl pH 7.5, 1 mM EDTA) to remove excess biotin. The dialysis buffer was changed at least thrice, once every 2 h. Following dialysis, the lysate was transferred to a 15-ml tube.

#### Immobilization on beads

The protein–RNA complexes were immobilized at low bead-surface density on streptavidin-coated beads (800 μl MyOne Streptavidin T1 beads, which is equivalent to 200 cm^2^ surface area). The advantages of immobilization on a solid surface include: (i) reduction of random intermolecular ligations between non-cross-linked oligonucleotides^58^, (ii) permit efficient buffer exchange and (iii) removal of non-physiologic interactions by stringent washes.

Eight hundred microlitres of MyOne T1 beads were washed thrice with PBST (PBS with 0.1% Tween-20), resuspended in 800 μl of the same buffer and transferred into the biotinylated lysate. The bead-lysate suspension was rotated at room temperature for 45 min. During this incubation, we prepared 200 μl of neutralized 25 mM IPB by adding equal molarity of DTT and incubating at room temperature for at least 30 min. The beads were immobilized using a magnetic stand and most of the supernatant was aspirated out, leaving behind 4 ml of the supernatant. The beads were resuspended in the leftover solution followed by the addition of 200 μl of neutralized IPB. IPB was used to saturate excess of unbound streptavidin after immobilization, which may interfere with subsequent step that involves biotin-tagged RNA linker. To remove the undesired RNAs non-covalently attached to proteins or via nonspecific protein–protein interactions^59^,^60^, the beads were washed three times with ice-cold denaturing washing buffer I (50 mM Tris-HCl pH 7.5, 0.5% lithium dodecyl sulfate, 500 mM lithium chloride, 7 mM EDTA, 3 mM EGTA, 5 mM DTT) with rotation at 4 °C for 5 min in every wash. Next, the beads were washed with ice-cold, high-salt wash buffer II (50 mM Tris-HCl pH 7.5, 1 M NaCl, 0.1% SDS, 1% IGEPAL CA-630, 1% sodium deoxycholate, 5 mM EDTA, 2.5 mM EGTA, 5 mM DTT), wash buffer III (1 × PBS, 1% Triton X-100, 1 mM EDTA, 1 mM DTT) and T4 polynucleotide kinase (PNK) wash buffer (20 mM Tris-HCl pH 7.5, 10 mM MgCl_2_, 0.2% Tween-20, 1 mM DTT); two times with each buffer, with a rotation for 5 min at 4 °C during the second wash.

#### Ligation of a biotin-tagged RNA linker

Next, a biotin-tagged RNA linker (5′- rCrUrArG/iBiodT/rArGrCrCrCrArUrGrCrArArUrG rCrGrArGrGrA -3′) was attached to the RNA's 5′-end. The biotin-tagged linker serves as a selection marker to enrich for the ligated RNAs; it also delineates a clear boundary to unambiguously split any sequencing read that covered a ligation junction. The 5′-end of the RNA linker was temporarily ‘blocked' from ligation, to avoid linker circularization or concatenation. This was achieved by synthesizing the linker with a 5′-OH group, which is incompatible with ligation but can be ‘re-activated' by phosphorylation. However, RNase I leaves 5′-OH end, which is incompatible for linker ligation; thus, we first phosphorylated the 5′-end with PNK, 3′-phosphatase minus (NEB). We did not use the wild-type T4 PNK due to its additional 3′-phosphatase activities, which modifies the 3′-ends of RNAs from 3′-P into 3′-OH, making them susceptible to self-ligation.

This was achieved by removing wash buffer and subsequently resuspending the beads in 100 μl of PNK reaction mixture (73 μl of RNase-free water, 10 μl of 10 × PNK buffer, 10 μl of 10 mM ATP, 5 μl of 10 U μl^−1^ T4 PNK (3′-phosphatase minus) (NEB), 2 μl of RNAsin Plus (Promega)) and incubating for 1 h at 37 °C with intermittent shaking at 1,200 r.p.m. for 5 s every 2 min. The beads were washed with wash buffer I, II, III and PNK, two times with each buffer, with rotation for 5 min at 4 °C in the second wash. The ice-cold washes were used to eliminate any leftover PNK, which may phosphorylate the RNA linker, inducing it to be potentially ligated to the 3′-end of RNAs. After wash buffer was removed, the biotin-tagged RNA linker was ligated to RNA 5′-ends by adding 160 μl RNA ligation reaction mixture, which contained 2 μl RNAsin Plus (Promega), 16 μl of 10 mM ATP, 16 μl of 10 × RNA ligase buffer, 16 μl of 1 mg ml^−1^ BSA, 30 μl of 20 μM biotin-labelled linker, 64 μl of 50% PEG8000 (NEB), 16 μl of 10 U μl^−1^ T4 RNA ligase 1 (NEB). Ligation was carried out at 16 °C overnight with intermittent shaking at 1,200 r.p.m. for 15 s every 2 min. BSA was added to enhance the activities of T4 RNA ligase and prevent bead aggregation. Polyethylene glycol (PEG) was used to enhance intermolecular ligation by increasing the concentrations of the donor and the acceptor ends^61^.

#### Proximity ligation

Next, the beads were washed twice with ice-cold wash buffer II, once with ice-cold wash buffer III and PNK wash buffer. To prepare for proximity ligation, we first dephosphorylated the RNA 3′-end using the 3′-phosphatase activities of T4 PNK, leaving a 3′-hydroxyl group^62^. After discarding wash buffer, the beads were mixed with 73 μl of RNase-free water, 20 μl of 5 × PNK buffer pH 6.5 (350 mM Tris-HCl pH 6.5, 50 mM MgCl_2_, 10 mM DTT), 5 μl of 10 U μl^−1^ T4 PNK (3′-phosphatase minus) (NEB), 2 μl of RNAsin Plus (Promega) and incubated for 20 min at 37 °C with intermittent shaking at 1,200 r.p.m. for 5 s every 2 min. The beads were washed once with PNK wash buffer and the 5′-end of the biotin-labelled linker was phosphorylated in 100 μl of PNK reaction mixture (73 μl of RNase-free water, 10 μl of 10 × PNK buffer, 10 μl of 10 mM ATP, 5 μl of 10 U μl^−1^ T4 PNK (3′-phosphatase minus) (NEB), 2 μl of RNAsin Plus (Promega)) for 1 h at 37 °C with intermittent shaking. Following phosphosrylation, the beads were wash twice in PNK wash buffer and proximity ligation was then performed under extremely diluted conditions in a 15-ml total volume reaction (8.9 ml of RNase-free water, 1.5 ml of 10 mM ATP, 1.5 ml of 10 × RNA ligase buffer, 75 μl of 20 mg ml^−1^ BSA (NEB), 25 μl of 1 M DTT, 2.25 ml of 100% dimethyl sulfoxide, 0.75 ml of 10 U μl^−1^ T4 RNA ligase 1 (NEB)), to minimize inter-complex ligations. The proximity ligation was carried out at 16 °C overnight with continuous rotation.

#### RNA purification and reverse transcription

The following day, ligation was stopped by adding EDTA to a final concentration of 25 mM and rotating for 15 min at 4 °C, to prevent inter-molecular ligation from happening as the beads were collected on the wall of the tube. The beads were washed once in PBST. We next eluted protein–RNA complexes from streptavidin beads twice in 100 μl of Elution Buffer (100 mM Tris-HCl pH 7.5, 50 mM NaCl, 10 mM EDTA, 1% SDS, 10 mM DTT, 2.5 mM D-biotin (Invitrogen)) by heating to 95 °C for 5 min. The resulting solutions were combined, mixed with 50 μl of 800 U ml^−1^ Proteinase (NEB) and incubated at 55 °C for 2 h. We topped up the mixture with RNase-free water to the final volume of 400 μl. RNAs were extracted in 400 μl of phenol:chloroform:isoamyl alcohol (125:24:1, pH 4.5) (Ambion) and incubation at 37 °C for 20 min with shaking at 1,000 r.p.m. The mixture was transferred into a 2-ml MaXtract high-density phase lock gel tube (Qiagen) and centrifuged at 16,000 *g* for 5 min at room temperature. Residual phenol was removed by adding 400 μl of chloroform to the same MaXtract tube and centrifugation at 16,000*g* for 5 min at room temperature. Following centrifugation, the aqueous phase was transferred into a new tube and RNAs were precipitated by adding 1:9 volume of 3 M sodium acetate pH 5.2, 1.5 μl of glycoblue (Ambion) together with 1 ml of 1:1 ethanol:isopropanol and incubating at −20 °C overnight. We pelleted the precipitated RNA by centrifugation at 21,000 *g* for 30 min at 4 °C. After discarding the supernatant, the pellet was washed twice with 80% ethanol and air dried until ethanol completely evaporated. The purified RNAs at this stage were a mixture of RNAs without linkers (RNA1 or RNA2), RNAs ligated with linkers but not proximity ligated with other RNAs (5′-linker–RNA2) and the desirable chimeric constructs in the form of 5′-RNA1–linker–RNA2. RNA1 can be depleted by selection of the biotin-tagged linker. We therefore depleted the non-informative 5′-linker–RNA2 as well in the next reaction with T7 exonuclease.

##### Removing biotin from terminal linkers (5′-linker–RNA2)

This was based on the RNase H activity of T7 exonuclease, which not only removes 5′-mononucleotides from duplex DNA but also exert exonucleolytic activity on the RNA strand from a RNA–DNA hybrid^63^. A complementary DNA oligonucleotide (5′- T*C*G*C*ATTGCATGGGCTACTAGCAT -3′, where * denotes the phosphorothioate bond to block its digestion by T7 exonuclease^64^) was annealed to the RNA linker, creating a double-stranded DNA–RNA hybrid between the RNA linker and the cDNA strand. The cDNA strand was designed so that after annealing the 5′-end of the RNA linker was recessed, while the 3′-end of the DNA strand was protruding. The annealed products were then treated with T7 exonuclease.

The RNA pellet was resuspended in 17 μl of RNase-free water, 4 μl of 10 × NEBuffer4 and 7 μl of 100 μM cDNA oligo. Annealing was performed by denaturing at 95 °C for 2 min and then slowly ramping down the temperature (at −0.1 °C s^−1^) to 25 °C. The annealed mixture was then mixed with 8 μl of 10 U μl^−1^ T7 exonuclease (NEB), 4 μl of 1 mg ml^−1^ BSA and incubated at 37 °C for 1 h. We removed the DNA oligonucleotides and any contaminating genomic DNA using TURBO DNase rigorous treatment: 44 μl of RNase-free water, 10 μl of 10 × TURBO DNase buffer and 6 μl of TURBO DNase (Invitrogen) was added, and the resulting mixture was incubated at 37 °C for 1 h. DNase-treated RNA was purified by phenol:chloroform extraction and ethanol precipitation as described above.

##### Removal of rRNAs by antibody-based depletion of RNA-–DNA hybrid (GeneRead rRNA Depletion Kit (Qiagen)) in ES-2 MEF samples

rRNA was removed according to the manufacturer's instructions with the following modifications. Instead of cleaning up depleted RNA by RNeasy MinElute spin columns, which will remove RNAs shorter than 200 nucleotides, we removed excess rRNA capture probes by rigorous DNase treatment. DNase-treated RNA was also purified by phenol:chloroform extraction and ethanol precipitation as described above.

##### RNA shearing

Following ethanol precipitation, RNA was fragmented into size range of 150–400 bp, optimal for sequencing by Illumina HiSeq, by using the RNase III fragmentation kit according to the manufacturer's protocol. Fragmented RNA was purified by 2.2 × SPRISelect beads (Beckman Coulter Genomics) and ethanol precipitated as described above.

##### Ligation with reverse transcription (RT) adapter

Next, the RNAs were ligated with a 3′-RT adapter (5′- /5rApp/AGATCGGAAGAGCGGTTCAG/3ddC/ -3′) that served as a primer for RT reaction. Following ethanol precipitation, the RNA pellet was resuspended in 20 μl of ligation reaction mixture: 1 μl RNAsin Plus (Promega), 2 μl of 10 × RNA ligase buffer, 7 μl of 20 μM pre-adenylated L3-App adapter, 8 μl of 50% PEG8000 (NEB), 2 μl of 200 U μl^−1^ T4 RNA ligase 2, truncated KQ (NEB). The reaction was incubated overnight at 16 °C.

##### Reverse transcription

Following ligation, RNA was purified by 2 × SPRISelect beads (Beckman Coulter Genomics) and eluted in RNase-free water. The following RT reaction is described for 2 μg of RNA and was scaled up accordingly for higher amount of RNAs. For each experiment or replicate, a different RT primer containing individual experimental barcode sequence was used. Each RT primer has the form of 5′- /5Phos/NNXXXXNNNNAGATCGGAAGAGCGTCGT**GgatcC**TGAACCGCTCTTCCGATCT -3′. According to this scheme, the first read of every sequencing read pairs contains a barcode that takes the configuration of NNNNXXXXNN (reverse complement of that from the RT primer), where the Ns are a random 6-nt barcode for removing PCR duplicates^19^,^50^,^51^,^52^. Any two pair-end reads with identical mapped locations and random barcodes would be counted as only one. The XXXX is a fixed 4-nt sample barcode for multiplexed sequencing (AGGT for ES-1, CGCC for ES-2, CATT for ES-indirect and CGCC for MEF). Any two 4-nt sample barcodes differ by three nucleotides, to avoid potential confusions from mutations or sequencing errors.

For cDNA synthesis, 9 μl of RNA was mixed with 1 μl 10 mM dNTPs and 1 μl of 50 μM RT primer. The mixture was heated at 65 °C for 5 min and snap-cooled in ice for at least 2 min. Four microlitres of 5 × First-Strand buffer (Invitrogen), 1 μl DTT, 0.1 M, 1 μl RNasin Plus and 1 μl of 10 mg ml^−1^ T4 gene 32 protein (NEB) were added. The resulting mixture was incubated at 50 °C for 2 min before adding reverse transcriptase enzyme to minimize mispriming. Next, we added 2 μl of 200 U μl^−1^ Superscript III reverse transcriptase (Invitrogen) to the solution. The RT reaction mixture was then incubated at 50 °C for 45 min, 55 °C for 20 min followed by 4 °C hold. Here, the heat inactivation of reverse transcriptase enzyme was omitted, to preserve the RNA–cDNA hybrids.

#### Biotin pull-down of chimeric RNA–DNA hybrids

Streptavidin–biotin affinity purification was used to enrich for chimeric RNA–DNA hybrids. This pull-down was carried out after the second RNA fragmentation and RT, to allow a substantial fraction of the sequencing read pairs to cover the RNA–linker or linker–RNA junctions, in one end of the read pair.

Specifically, 50 μl of Myone C1 beads (Invitrogen) was prepared by washing twice with 1 × Tween B&W buffer (5 mM Tris-HCl pH 8.0, 0.5 mM EDTA, 1 M NaCl, 0.05% Tween) and once with 1 × B&W buffer (5 mM Tris-HCl pH 8.0, 0.5 mM EDTA, 1 M NaCl). The beads were then resuspended with 100 μl of 2 × B&W buffer (10 mM Tris-HCl pH 8.0, 1 mM EDTA, 2 M NaCl). The RT mixture was topped up with RNase-free water to the final volume of 100 μl before combining with 100 μl C1 bead suspension and incubated at RT for 30 min with rotation. The beads were reclaimed and washed thrice with 1 × B&W buffer before transferring into a new tube, followed by washing once with TE buffer pH 8.0. Next, the cDNA strand was released from streptavidin beads by completely digesting the RNA strand in 50 μl RNase H elution mixture (39.5 μl of RNase-free water, 5 μl 10 × RNase H reaction buffer, 0.5 μl 10% Tween-20, 5 μl of 5 U μl^−1^ RNase H (NEB)) for 1 h at 37 °C. The beads were collected on the tube wall using a magnetic concentrator and the supernatant was collected in a new tube for subsequent manipulations. We inactivated RNase H by heating at 70 °C for 20 min. cDNA was purified by 2.2 × SPRISelect beads (Beckman Coulter Genomics) (v/v).

#### Construction of sequencing library

Considering the ultraviolet-induced cross-link site sometimes stalls RT, resulting in truncated cDNAs that lack the 5′-adapter^65^, we adopted a circularization strategy that allowed for constructing sequencing libraries even from truncated cDNAs^62^ (Supplementary Fig. 1). The RT primer contained the adapter regions to prime PCR amplification by Illumina PE PCR Forward Primer 1.0 (5′- AATGATACGGCGACCACCGAGATCTACACTCTTTCCCTACACGACGCTC TTCCGATCT -3′) and PE PCR Reverse Primer 2.0 (5′- CAAGCAGAAGACGGCATACGAGATCGGTCTCGGCATTCCTGCTGAACCGCTCTTCCGATCT -3′), flanking a BamHI restriction site and a sequencing barcode.

##### Circularization

cDNA was circularized by CircLigase II (Epicentre). Briefly, cDNA was eluted from SPRISelect beads in 20 μl CircLigase reaction mixture (12 μl of sterile water, 2 μl of CircLigase II 10 × reaction buffer, 1 μl of 50 mM MnCl_2_, 4 μl of 5 M Betaine, 1 μl of 100 U μl^−1^ CircLigase II (Epicentre)) and incubated for 2 h at 60 °C. CircLigase II was inactivated by incubating the reaction at 80 °C for 10 min.

##### Relinearization

A cDNA oligo was annealed to the RT primer, generating a short double-stranded region suitable for BamHI restriction. This strategy also prevents BamHI activities on other endogenous BamHI restriction sites. Next, BamHI were applied, creating linear cDNAs with adapters at both 5′- and 3′-ends to prime subsequent PCR amplification. Next, oligo annealing mixture (43 μl water, 6 μl 10 × FastDigest Buffer (Fermentas), 5 μl 20 μM Cut_oligo (5′- GTTCA**GGATCC**ACGACGCTCTTCAAAA/3InvdT/ -3′) was added into the CircLigase II reaction. Annealing was carried out by heating to 95 °C for 2 min, followed by 71 cycles of 20 s each, starting from 95 °C and decreasing the temperature by 1 °C after every cycle down to 25 °C and holding at 25 °C. Six microlitres of FastDigest BamHI (Fermentas) was added and incubated at 37 °C for 30 min. Re-linearized cDNA was purified by 2 × SPRISelect beads (Beckman Coulter Genomics) (v/v) and eluted in nuclease-free water.

##### First PCR pre-amplification and size selection

Single-stranded cDNA was first pre-amplified by PCR using a truncated version of PCR primers (forward primer DP5, 5′- CACGACGCTCTTCCGATCT -3′ and reverse primer DP3, 5′- CTGAACCGCTCTTCCGATCT -3′) with small number of cycles (six cycles). We found that the final libraries were less prone to be contaminated with undesirable smaller size fragments (primer–dimers, products which contain only the barcode and/or RNA linker) by doing size selection at this stage.

Six cycles of PCR were performed in a 40-μl reaction, which contained 20 μl of NEBNext High-Fidelity 2 × PCR Master Mix (NEB), 0.625 μM of each DP5/DP3 primer using the following temperatures: 1 cycle of initial denaturation at 98 °C for 30 s; 6 cycles of amplification with 98 °C for 10 s, 65 °C for 30 s, 72 °C for 30 s; followed by final extension at 72 °C for 5 min; and hold at 4 °C. The PCR product was purified by 1.8 × SPRISelect beads (v/v) and size-selected using E-gel EX 2% Agarose gels (Invitrogen). The DNA fragments between 150 bp and 350 were excised from the gel and purified using MinElute gel extraction kit (Qiagen).

##### rRNA removal by duplex-specific nuclease (DSN) approach^66^

(ES-1, ES-indirect). To reduce rRNA cDNAs from ES-1 and ES-indirect library, we also pre-amplified ss-cDNA using the truncated PCR primer DP5/DP3. However, the PCR cycle number was increased until we could obtain 80–100 ng of cDNA after purification by 1.8 × SPRISelect beads (Beckman Coulter Genomics) (v/v). We skipped the size selection by agarose gel, as this would largely reduce the amount of DNA. The eluted DNA from SPRISelect beads was mixed with 4.5 μl hybridization buffer (2 M NaCl, 200 mM HEPES pH 8.0) and sterile water (if necessary) to a final volume of 18 μl. The resulting mixture was denatured at 98 °C for 2 min and re-annealed at 68 °C for 5 h on a thermal cycler. Although the reaction mix tube was still in the thermal cycler, we added 20 μl of 68 °C-preheated 2 × DSN buffer (Axxora) to the reaction mix, mixed well by pipetting up and down ten times and incubated the reaction for 10 min at 68 °C. Two microlitres of 1 U μl^−1^ DSN enzyme (Axxora) was added, mixed and incubated at 68 °C for 25 more minutes. We stopped the reaction by adding 40 μl of 2 × DSN stop solution (Axxora) to the reaction mix tube, mixing well and transferred the tube to ice. The reaction mixture was then purified using 1.8 × SPRISelect beads.

##### Final PCR amplification

We performed PCR amplification of DNA produced from previous steps using full-length PCR primer PE 1.0 and 2.0 (Illumina). The number of PCR cycles was carefully titrated by running pilot PCRs with small aliquots of DNA to avoid overamplification. We purified the PCR products by 1.8 × SPRISelect beads (v/v) and size-selected fragments between 250 and 550 (120–420 bp insert plus ∼130 bp, the combined length of Illumina PE 1.0/2.0). Final libraries were quantified by Qubit (Invitrogen) and quantitative PCR, quality-checked by Bioanalyzer (Agilent Technologies) and submitted for paired-end sequencing on Illumina HiSeq platform.

### Oligonucleotide sequences used in MARIO

The custom-designed RNA and DNA oligonucleotides used in the procedure are as follows:

Biotinylated RNA linker (RNase-free HPLC-purified from IDT):

5′- rCrUrA rG/iBiodT/rA rGrCrC rCrArU rGrCrA rArUrG rCrGrA rGrGrA -3′

cDNA strand with RNA linker (RNase-free HPLC-purified from Sigma):

5′- T*C*G*C*ATTGCATGGGCTACTAGCAT -3′

Pre-adenylated RT adapter (RNase-free HPLC-purified from IDT):

5′- /5rApp/AGATCGGAAGAGCGGTTCAG/3ddC/ -3′

RT primers (adapted from ref. 62) (RNase-free HPLC-purified from Sigma):

RT Primer for the ES-1 sample:

5′- /5Phos/NNAGGTNNNAGATCGGAAGAGCGTCGT**GgatcC**TGAACCGCTCTTCCGATCT -3′

RT Primer for the ES-2 and MEF samples (sequenced on different lanes):

5′- /5Phos/NNCGCCNNNNAGATCGGAAGAGCGTCGT**GgatcC**TGAACCGCTCTTCCGATCT -3′

RT Primer for the ES-indirect sample:

5′- /5Phos/NNCATTNNNNAGATCGGAAGAGCGTCGT**GgatcC**TGAACCGCTCTTCCGATCT -3′

Cut_oligo (HPLC-purified from IDT):

5′- GTTCA**GGATCC**ACGACGCTCTTCAAAA/3InvdT/ -3′

BamHI restriction site is highlighted

Truncated PCR Forward Primer DP5 (HPLC-purified from IDT):

5′- CACGACGCTCTTCCGATCT -3′

Truncated PCR Reverse Primer DP3 (HPLC-purified from IDT):

5′- CTGAACCGCTCTTCCGATCT -3′

Illumina PE PCR Forward Primer 1.0 (PAGE-purified from Sigma):

5′- AATGATACGGCGACCACCGAGATCTACACTCTTTCCCTACACGACGCTCTTCCGATCT -3′

Illumina PE PCR Reverse Primer 2.0 (PAGE-purified from Sigma):

5′- CAAGCAGAAGACGGCATACGAGATCGGTCTCGGCATTCCTGCTGAACCGCTCTTCCGATCT -3′

### Single-molecule RNA-FISH

Oligonucleotides were synthesized with a biotin attached to their 5′-end (IDT). Labelling was achieved by incubation of oligonucleotides and dyes (Alexa 555 or qDots, Invitrogen) coupled with streptavidin at room temperature for 30 min at a ratio of 0.5 μM of oligonucleotides per 1 μM of dye.

ES cells were seeded on glass-bottom micro-chamber (1.5, Lab Teck) previously coated with poly-D-lysine (5 μM, Sigma) and laminin (0.01 mg μl^−1^, Sigma). Following incubation for 2 h, cells were washed in nuclease-free PBS and permeabilized with methanol at −20 °C. smRNA-FISH experiments were conducted using a modified version of an established protocol^67^. Hybridizations were carried with ∼15 μM of oligonucleotides in hybridization buffer for 30 min at 40 °C. Excess of probes and dyes was removed by two washes (SSC 2 × , formamide 10%) at 37 °C for 30 min. The cells were then imaged in SSC 2 × buffer (pH 7.5).

Wide-field fluorescence imaging was conducted in an Olympus IX83 inverted microscope equipped with appropriate cubes for the dyes used (Supplementary Table 4, Chroma) and × 60 oil-immersion objective (numerical aperture=1.4, Olympus). Images were captured with ORCA-R2 charge-coupled device camera (Hamamatsu) at intervals of 0.2 μm on the *z* axis.

Following imaging, raw image stacks were processed in ImageJ^68^, first by applying the Laplacian of Gaussian filter^69^, second by counting the three-dimensional-rendered signal spots^70^ at incrementing intervals of 500 fluorescence arbitrary units. The number of spots in each image stack was obtained at the threshold interval of three consecutive equal counts. We observed that by selecting fluorescence intensities beyond this plateau one would have reduced the number of spots identified in the order of one unit, with the potential increase in false-negative spots. In addition to the counts, we collected data for the spot's respective *x*–*y*–*z* centre of mass.

### The computational pipeline (MARIO tools)

MARIO tools is a package of command-line tools for analyses of MARIO data. It is written in Python and R, and is version controlled by GitHub. The full documentation of the MARIO tools software is available at http://mariotools.ucsd.edu. The pipeline takes pair-end sequencing reads as input (Supplementary Fig. 26A). The oligonucleotide sequences of the RNA linker and the sample barcodes used for multiplexed sequencing should also be provided to the pipeline. The main outputs include: (1) a parsed cDNA library, including the list of chimeric cDNAs in the form of RNA1–Linker–RNA2 (see the final products in Supplementary Figs 1 and 26C); (2) the genomic locations of RNA1 and RNA2 of every chimeric cDNA (Supplementary Fig. 26D); and (3) interacting RNA pairs inferred from statistical enrichment of chimeric cDNAs (Supplementary Fig. 26E). The major analysis steps of MARIO tools are as follows: (1) removing PCR duplicates; (2) assigning multiplexed sequencing reads into corresponding experimental samples; (3) recovering the cDNAs in the sequencing library; (4) parsing the chimeric cDNAs; (5) mapping to the genome; (6) identifying interacting RNA pairs; and (7) identifying RNA interaction sites. Detailed documentation of MARIO tools is available at http://mariotools.ucsd.edu

### Binding energies between RNA interaction sites

The binding energies between two RNA interaction sites were calculated by the DuplexFold programme from RNAstructure version 5.6 (ref. 43). The base paring between two interaction sites was determined by MiRanda version 3.3a.

### Conservation levels of RNA interaction sites

For every read pair in the RNA1–Linker–RNA2 category (output of Step 4 from MARIO tools), we obtained the PhyloP conservation scores^45^ of two 1,000-bp genomic regions, one centred at the ligation junction of RNA1–Linker and the other centred at the ligation junction of Linker–RNA2. The average PhyloP scores of all the RNA1–Linker–RNA2-type read pairs were plotted. As a control, we obtained average PhyloP scores from the same number of random genomic regions of the same lengths.

### Detecting read pairs

Starting from the RNA1–Linker–RNA2 type of read pairs (output of ‘Step 6. Selection and extraction of desired RNA–RNA interactions and reverse transcription' from MARIO tools), we applied the following filters to identify the pair-end reads generated from self-interacting RNAs. We removed those read pairs mapped to two different genes. If a read pair mapped to the same gene, we also removed those pairs that: (1) did not contain any fraction of the linker sequence; (2) the forward and the reverse reads mapped to opposite strands within 2,000 bp; (3) the read mapped to plus strand has smaller coordinates than the read mapped to minus strand in the genome within the pair. This step minimizes the inclusion of any intact (continuous) RNA fragment in the structural analysis.

### RNA folding and secondary structure prediction

Structural information of the RNAs with known or generally accepted structures was downloaded from fRNAdb database v3.4 in DOT format (graph description language). We drew figures from the DOT files using the command line version of VARNA Applet version 3.9. For the RNAs without structural information in fRNAdb, we predicted their secondary structures based on the sequence using the ‘Fold' programme in RNAstructure version 5.6^43^.

### Data availability

Data that support the findings of this study have been deposited in Gene Expression Omnibus with the accession number GSE61489.

## Additional information

**Accession codes:** Data that support the findings of this study have been deposited in Gene Expression Omnibus with the accession number GSE61489.

**How to cite this article:** Nguyen, T. C. *et al*. Mapping RNA–RNA interactome and RNA structure *in vivo* by MARIO. *Nat. Commun.* 7:12023 doi: 10.1038/ncomms12023 (2016).

## Supplementary Material

Supplementary Information Supplementary Figures 1-28, Supplementary Tables 1-8, Supplementary Notes 1-5 and Supplementary References
