# Supplementary material for: Mapping RNA–RNA interactome and RNA structure in vivo by MARIO
Source: Nat Commun. 2016 Jun 24;7:12023. doi: 10.1038/ncomms12023 (PMC4931010; doi:10.1038/ncomms12023)
Supplement: Supplementary Information — Supplementary Figures 1-28, Supplementary Tables 1-8, Supplementary Notes 1-5 and Supplementary References [file ncomms12023-s1.pdf]

Supplementary figures

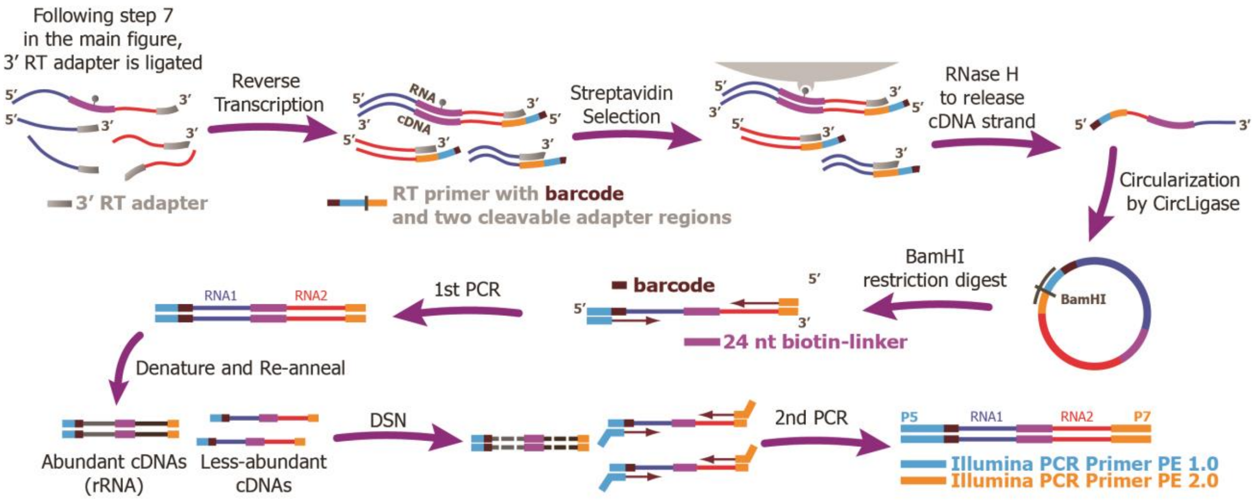

Supplementary Figure 1. A circularization strategy for construction of sequencing libraries. This figure elaborates Step 8 of the MARIO procedure. A reverse transcription (RT) adaptor (grey) was attached to the 3' end of the RNAs. This RT adaptor was complementary to a fraction (orange) of a RT primer, which also contained an adaptor for the P5 sequencing primer (blue), a 10nt barcode (maroon), and a BamHI restriction site (black vertical line). After circularization, a DNA oligo (black) containing the BamHI site was hybridized to the RT primer region, providing a double stranded substrate for BamHI digestion. Linearized ss-cDNAs were amplified by truncated PCR primers DP5 and DP3 to obtain ~100ng of ds-cDNAs, which were then denatured and reannealed. Duplex-specific nuclease (DSN) was used to deplete cDNAs that were originated from rRNAs. DSN selectively removes the ds-cDNAs that were formed earlier during the reannealing process. The cDNAs originated from rRNAs should be more abundant and therefore reanneal faster than the other cDNAs. The DSN-treated products were PCR-amplified again by Illumina PCR primers PE 1.0 and 2.0 to generate libraries suitable for sequencing. DSN based rRNA removal was applied to ES-1. ES-2 was subjected to an antibody based rRNA removal strategy that is not depicted in this figure.

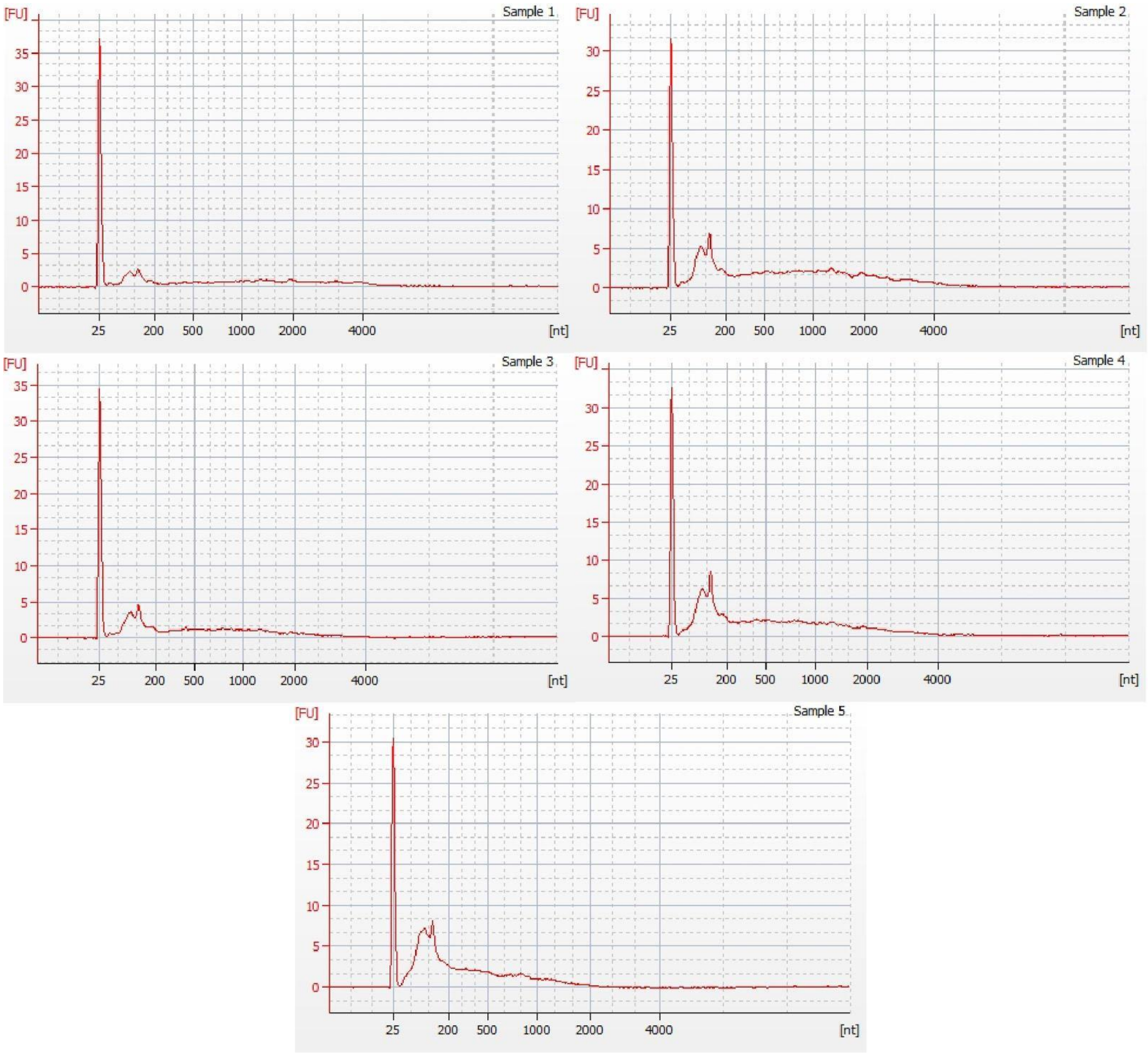

Supplementary Figure 2. Optimizing RNase I concentration for the first fragmentation. RNAs were purified from RNaseI-treated ES cell lysate by adding equal volume of 2x Proteinase K buffer (100 mM Tris-HCl pH 7.5, 100 mM NaCl, 2% SDS, 20 mM EDTA) and 1:5 volume of 20 mg/ml Proteinase K (NEB) and incubating at 55°C for 2 hours before phenol:chloroform treatment and ethanol precipitation. RNase I quantity per ml of cell lysate were: 0U (Sample 1), 2.5U (Sample 2), 3.3U (Sample 3), 5U (Sample 4), and 12.5 (Sample 5). The concentration of 5.0U RNase I/ml lysate that produced 500-1000nt RNA fragments (Sample 4) was chosen for MARIO Step 2.

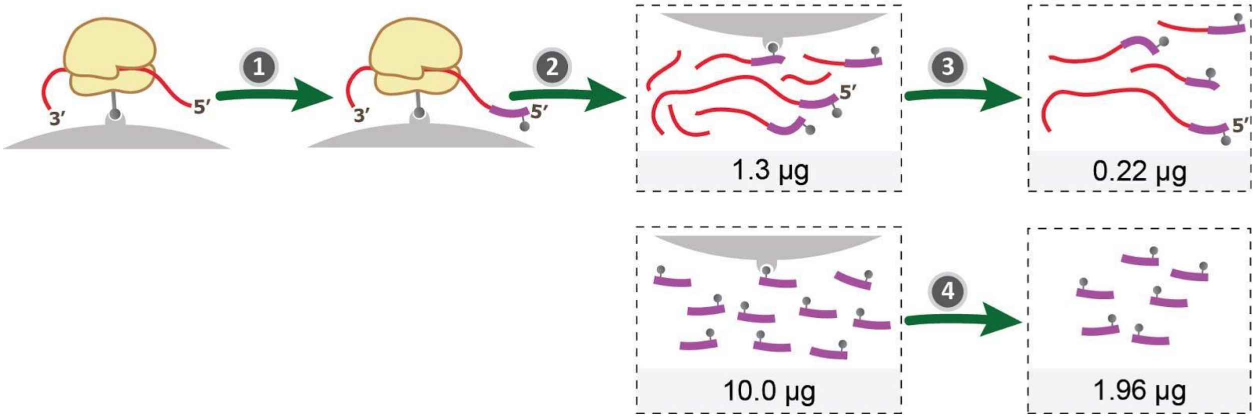

Supplementary Figure 3. Testing the efficiency of linker ligation on beads. Immobilized RNAs were digested with RNase I and then ligated with the biotin-labeled RNA linkers (1). After ligation and proteinase K digestion to remove the proteins, RNAs were purified and quantified (1.3µg) (2). The purified RNAs were then subjected to streptavidin-biotin pulldown to select for RNAs ligated to the biotin-labeled linker (3). After washing and eluting RNAs that were bound to streptavidin beads and ethanol precipitated, 0.22µg of RNA was collected. In parallel, the biotin-labeled RNA linkers were subjected to the same streptavidin-biotin pulldown, elution and ethanol precipitation (4). Assuming that the efficiencies of biotin pulldown, RNA elution and ethanol precipitation in Steps 3 and 4 were the same, about 19.6% (1.96µg / 10.0µg), we estimated the ligation efficiency as (0.22µg/19.6%)/1.3µg = 86%.

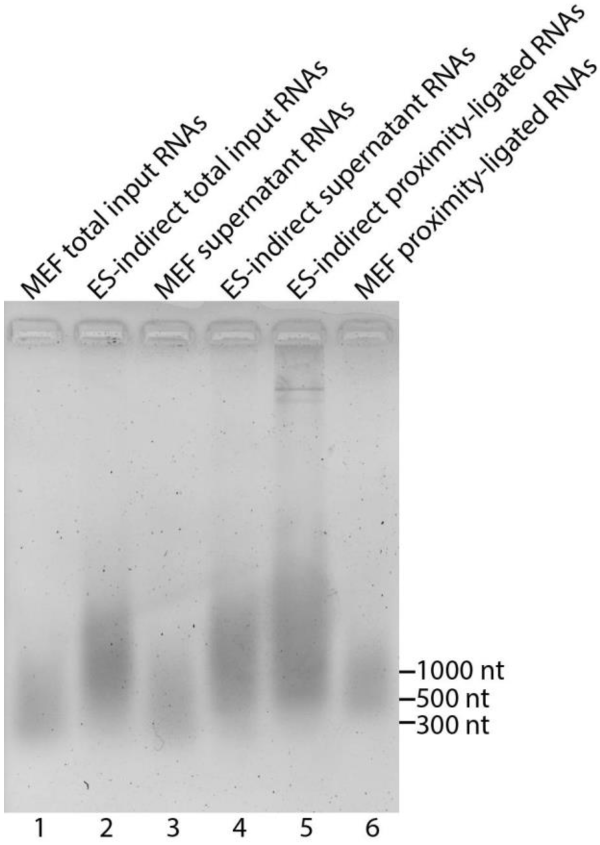

Supplementary Figure 4. RNA size distributions at different steps of the MARIO procedure. Only the ES-indirect and the MEF samples had sufficient intermediate products left for this retrospective analysis. Size distributions of RNAs in the lysates of MEF (Lane 1) and ES-indirect (Lane 2) before being tethered onto streptavidin beads, in the supernatant after immobilization (Lanes 3 and 4), and immobilized on beads after proximity ligation (ES-indirect: Lane 5, MEF: Lane 6). RNA was denatured in 2X RNA loading dye (NEB) at 70°C for 5 minutes, run on 1.5% Native Agarose gel and stained with SYBR Gold (Invitrogen).

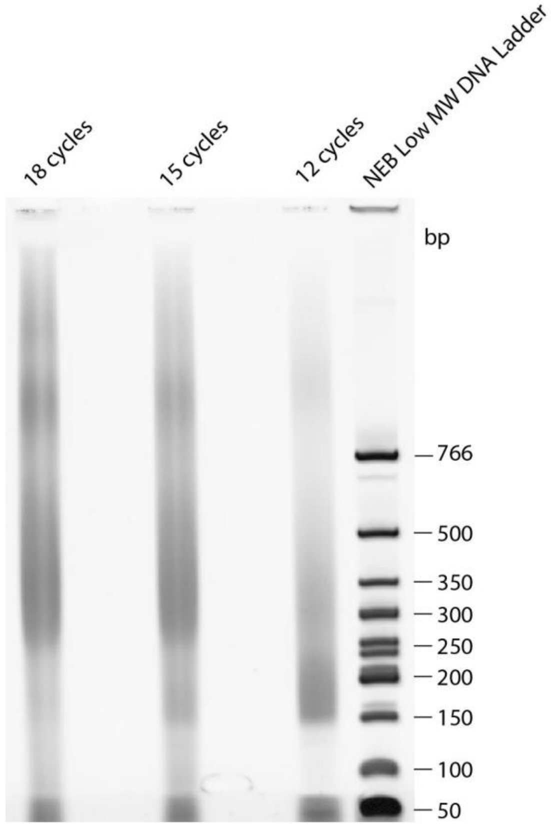

Supplementary Figure 5. Optimization of the number of PCR cycles for construction of sequencing library. In Step 8 of the MARIO procedure, single-stranded cDNAs of the ES-1 sample were pre-amplified with 12 cycles of PCR using a truncated form of Illumina PCR sequencing primers (DP5, DP3). The PCR products were purified with 1.8x SPRISelect beads, which produced 86 ng of double-stranded DNAs before the depletion of the cDNA synthesized from rRNA by duplex-specific nuclease. One µl aliquots from a total of 22 µl of rRNA-depleted double-stranded cDNAs were amplified with various PCR cycle numbers (12, 15, 18) using NEBNext High-Fidelity 2X PCR Master Mix (NEB) and Illumina PE Primer 1.0 and 2.0. The PCR products were assayed on 6% TBE PAGE gel and stained with SYBR Gold (Invitrogen). Based on the gel result, 18 µl of original rRNA depleted double-stranded DNAs were then amplified with 11 cycles of PCR to generate the sequencing library.

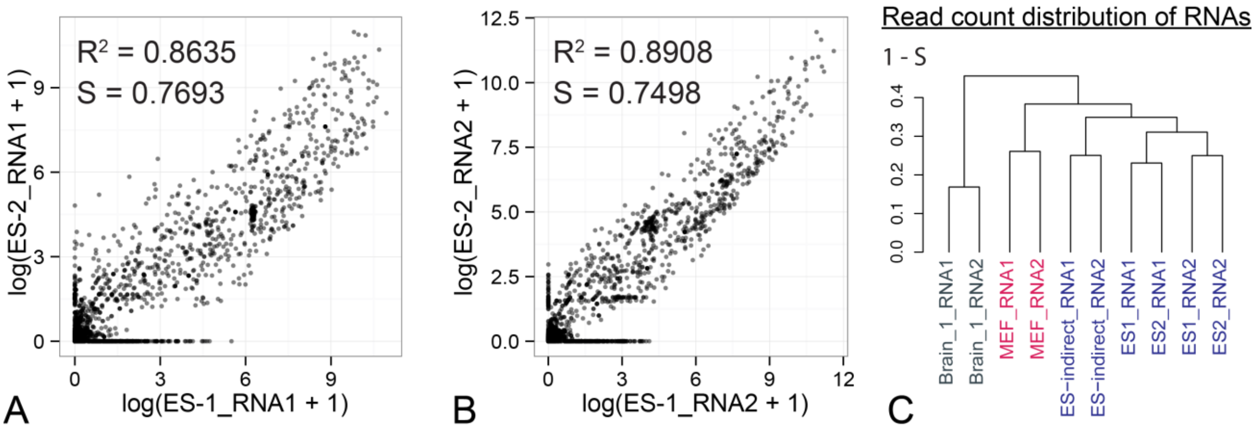

Supplementary Figure 6. Comparison of MARIO libraries. (A-B) The read fragment at the 5’ end (RNA1) and the 3’ end (RNA2) of the linker were separately analyzed as two RNA-seq experiments. Scatter plots of the read count distribution (FPKM) of all known RNAs between ES-1 and ES-2 samples at log scale. R: Pearson correlation. S: Spearman correlation. (C) Hierarchical clustering of FPKMs of each sample.

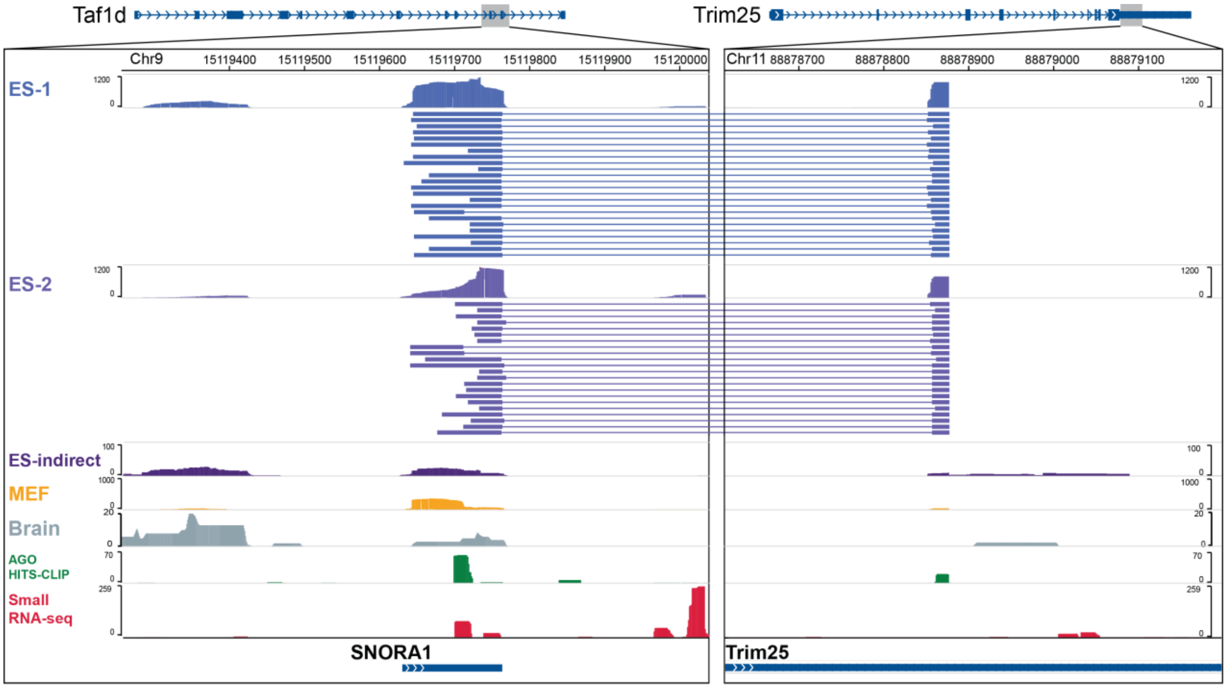

Supplementary Figure 7. MARIO data mapped to the genome. Ligation of *Trim25* and *Snora1* RNAs was supported by multiple pair-end reads in ES-1 and ES-2 libraries. Ago CLIP-seq: AGO HITS-CLIP of mouse ES cells (GEO: GSM622570). Small RNA-seq: sequencing of small RNAs with a 3’ hydroxyl group resulting from enzymatic cleavage (GEO: GSM945907).

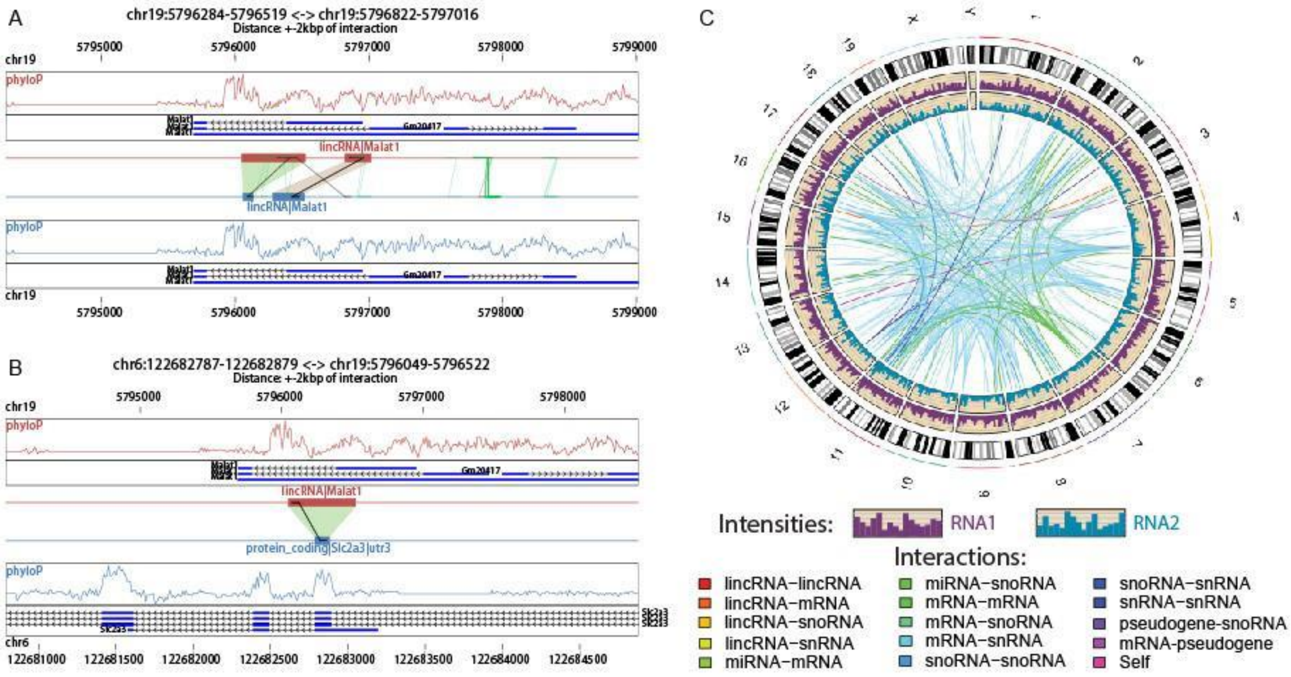

Supplementary Figure 8. Visualization capabilities of MARIO. (A-B) Detailed views of RNA interaction sites in intra-RNA (A) and inter-RNA (B) interactions. The two genomic regions containing the two interacting RNAs were plotted in parallel (panel B). Each RNA1-Linker-RNA2 type of chimeric RNA was plotted with the RNA1 and the RNA2 fragments mapped to the respective genomic regions, connected by an oblique line representing the linker. Red and blue blocks represent the “peaks” of overlapping MARIO reads, which were candidate RNA interaction sites. A semi-transparent polygon connecting two RNA interaction sites (red or blue blocks) represents a strong interaction. (C) A global view of the RNA-RNA interactions. The read densities of the RNA1 and the RNA2 fragments were shown in purple and blue tracks, respectively, inside chromatin cytoband ideogram. Each identified RNA-RNA interaction was shown as a curve connecting the genomic loci of the two RNAs, and colored by the types of the interacting RNAs.

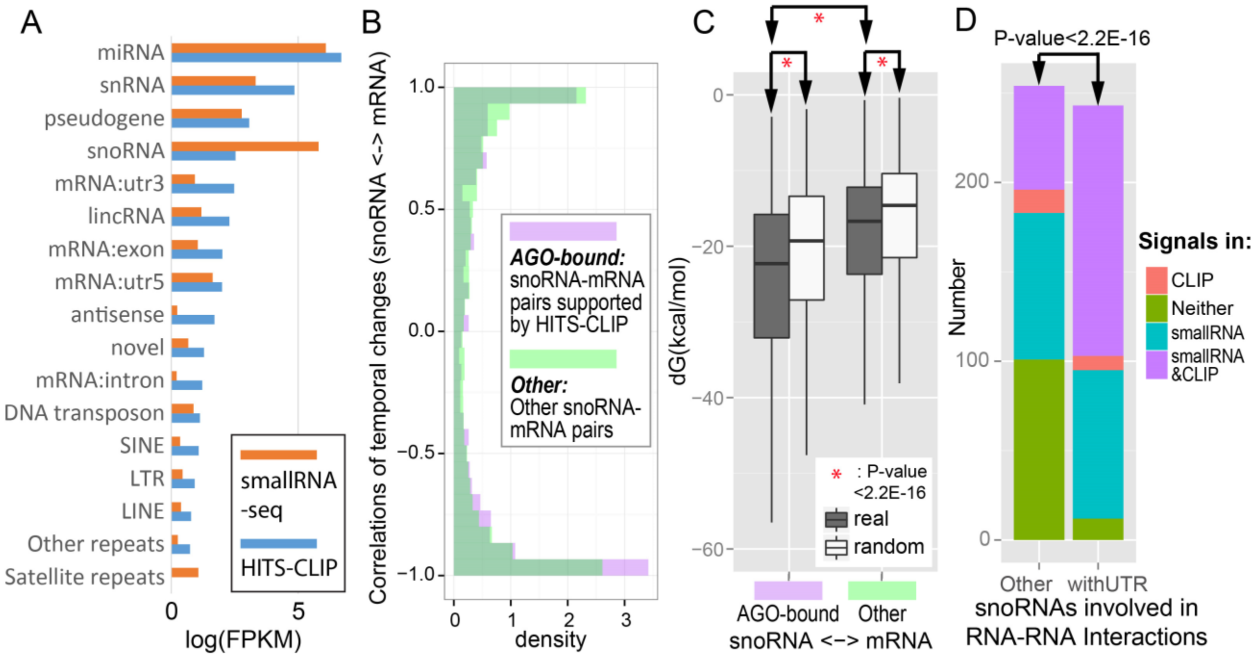

Supplementary Figure 9. snoRNAs with miRNA-like interactions. (A) Comparison of MARIO with smallRNA-seq (GSM945907) and AGO HITS-CLIP (GSM622570). The average FPKM of each type of MARIO identified interaction participating RNAs in smallRNA-seq (orange bars) and AGO HITS-CLIP (blue bars) is shown in log scale. The miRNAs and snoRNAs in MARIO identified interactions were enriched in both smallRNA-seq and AGO HITS-CLIP. (B) Distribution of the correlations of gene expression between every pair of interacting snoRNA and mRNA. The interacting snoRNA-mRNA pairs bound by AGO (purple, defined by AGO HITS-CLIP) were more negatively correlated than the pairs not bound by AGO (green) (p-value=4.18^{-5}, Kolmogorov-Smirnov Test). (C) Base pairing of the interacting RNAs as measured by hybridization energy. The snoRNA-mRNA pairs bound by AGO (intersected with AGO HITS-CLIP, left) exhibited stronger hybridization energies than those not bound by AGO (right) (p-value < 2.2^{-16}, Wilcoxon signed-rank test). All these interactions (grey boxes) exhibited stronger hybridization energies than those with randomly shuffled sequences (white boxes). (D) The snoRNAs interacted with the UTR regions of mRNAs were enriched in smallRNA-seq and AGO HITS-CLIP. The total number of interactions (y axis) between snoRNAs and mRNA coding regions (left) is decomposed into those detected in both smallRNA-seq and HITS-CLIP (purple), in smallRNA-seq only (blue), in HITS-CLIP only (pink), and in neither datasets (green). The interactions between snoRNAs and mRNA UTRs were similarly decomposed (right).

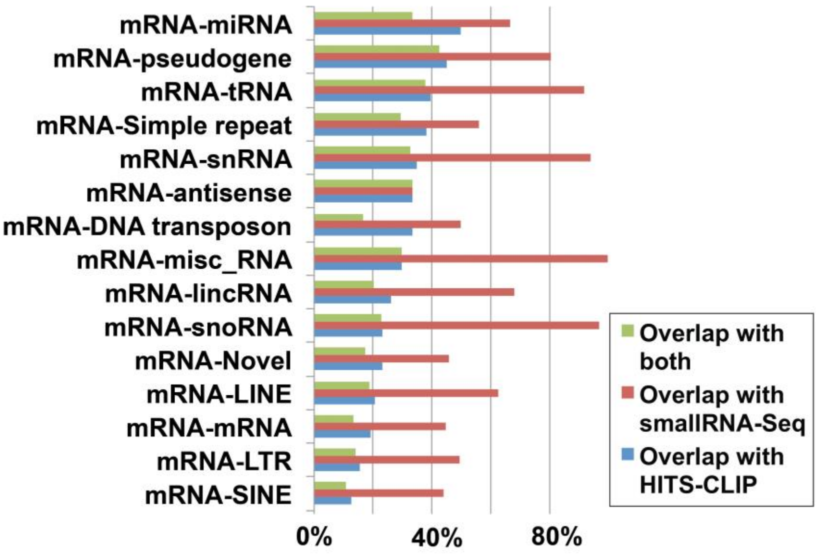

Supplementary Figure 10. Comparisons between MARIO and smallRNA-seq and AGO HITS-CLIP. The percentages of MARIO identified interactions that intersected with smallRNA-seq (red bars), AGO HITS-CLIP (blue bars), and both (green). The MARIO interactions were categorized by the types of participating RNAs, and the categories were ranked by the overlap with HITS-CLIP. misc_RNA: miscellaneous RNA, including RNase_MRP, 7SK RNA and others. Novel: unannotated RNA.

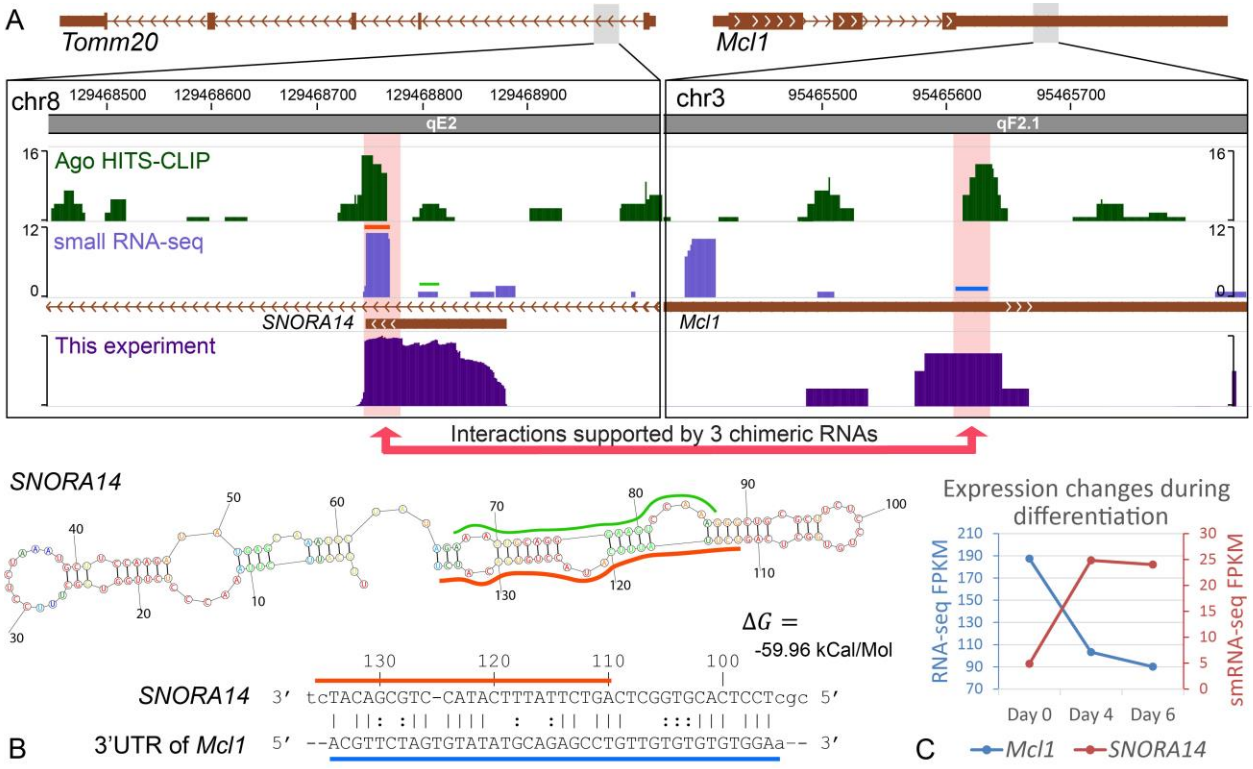

Supplementary Figure 11. Interaction between enzymatically processed *SNORA14* and *Mcl1* mRNA. (A) The MARIO identified interaction site on *SNORA14* intersected with smallRNA-seq, suggesting the *SNORA14* RNA was enzymatically processed into a shorter form (orange bar on the purple peak, 2nd row). This enzymatically processed small RNA corresponded to the end of the *SNORA14* hairpin (orange bar on the secondary structure), as well as the antisense to 3' UTR of *Mcl1* (orange bar in (B)). (C) Expression levels of the small RNA processed from *SNORA14* RNA (red) and *Mcl1* mRNA (blue) during the differentiation of ES cells to endomesoderm cells.

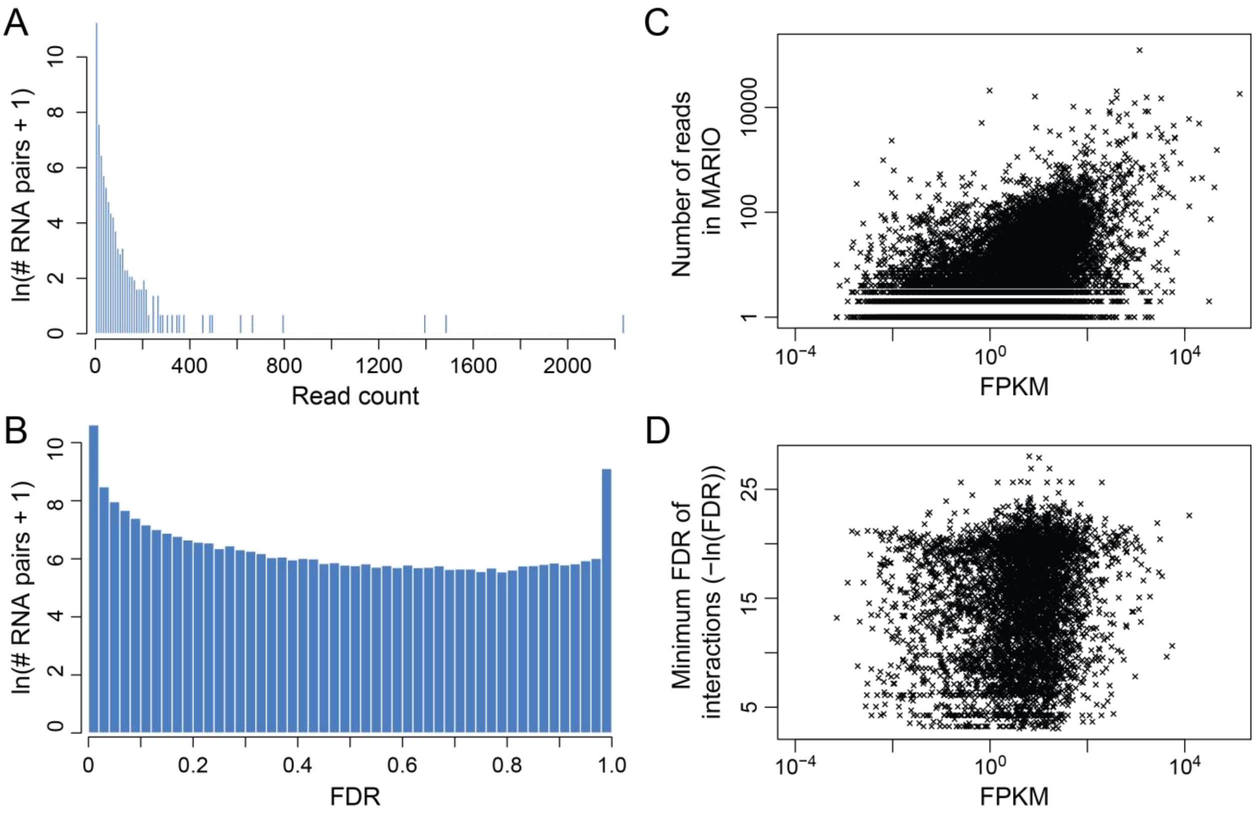

Supplementary Figure 12. Distributions of read counts and FDRs and relationships with gene expression. (A) Distribution of the number of read pairs mapped to every pair of RNAs. (B) Distribution of FDRs of every RNA pair from Fisher's Exact Test. (C) Scatter plot of the number of MARIO reads mapped to each RNA (y axis) and FPKM (x axis). (D) Scatter plot of the FPKM of each RNA (x asix) and the FDR (in minus log) of strongest interaction involving this RNA. The FPKM values were obtained by mapping raw reads from mouse ENCODE dataset ENCSR000CWC (paired-end RNA-Seq from E14 mouse ES cells) with bowtie2-2.2.4 against mm9, followed by processing with cufflink 2.2.1. All the genes with unique Ensembl IDs that were found in both ENCSR000CWC data and our mouse ES cell data are included in panels (C) and (D).

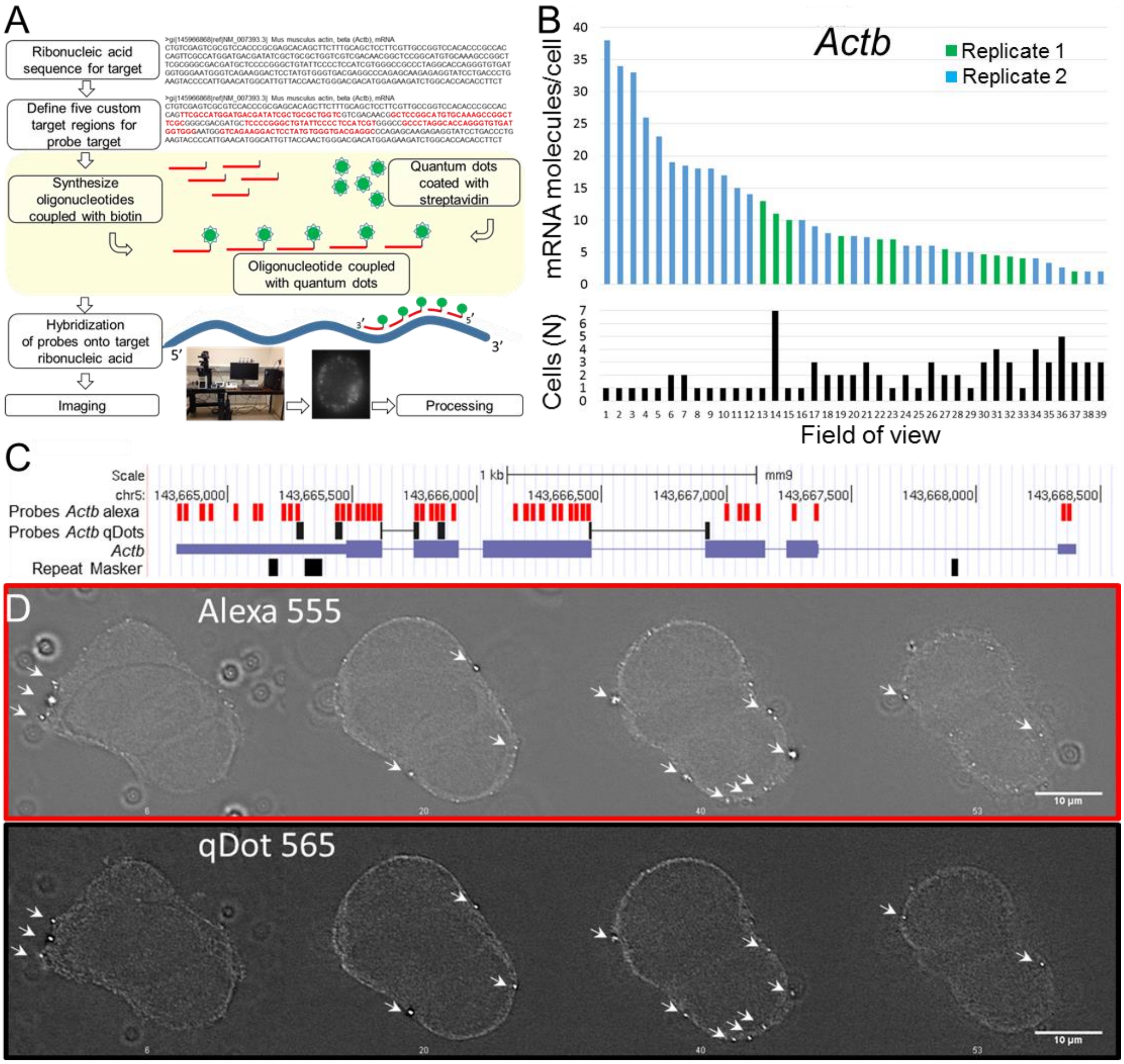

Supplementary Figure 13. Detection of RNA molecules with smRNA-FISH. (A) Scheme of single molecule RNA-FISH with probes labeled with quantum dots. (B) Distribution of *Actb* mRNA molecules in 82 single ES cells, in 39 fields of view, from two independent experiments (Replicates 1 and 2). (C) Genomic positions for smRNA-FISH probes labeled with organic dyes (red) and probes labeled with qDots (black) for the same gene (*Actb*). (D) Co-localization of signals detected (arrows) from probes labeled with organic (Alexa 555) and inorganic (qDot 565) dyes.

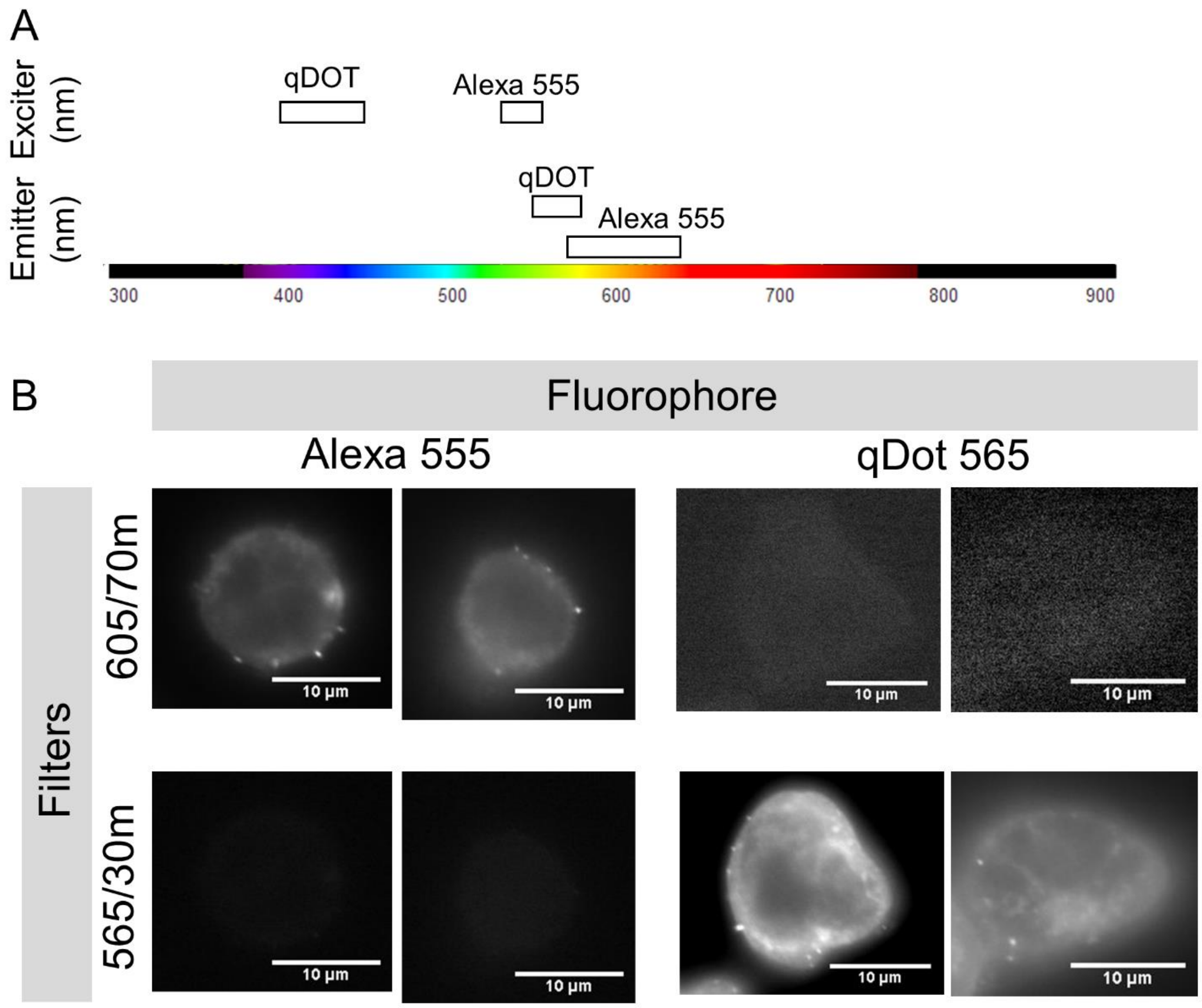

Supplementary Figure 14. Distinctions of Alexa 555 and qDot 565 excitation and emission. (A) The excitation wave lengths of qDots and Alexa 555 were distinct (Exciter lane). (B) RNA-FISH signals of Alexa 555 and qDot 565 acquired with corresponding and exchanged emission filters. The smRNA-FISH of the *Actb* mRNA were carried out as described in Supplementary Figure 13.

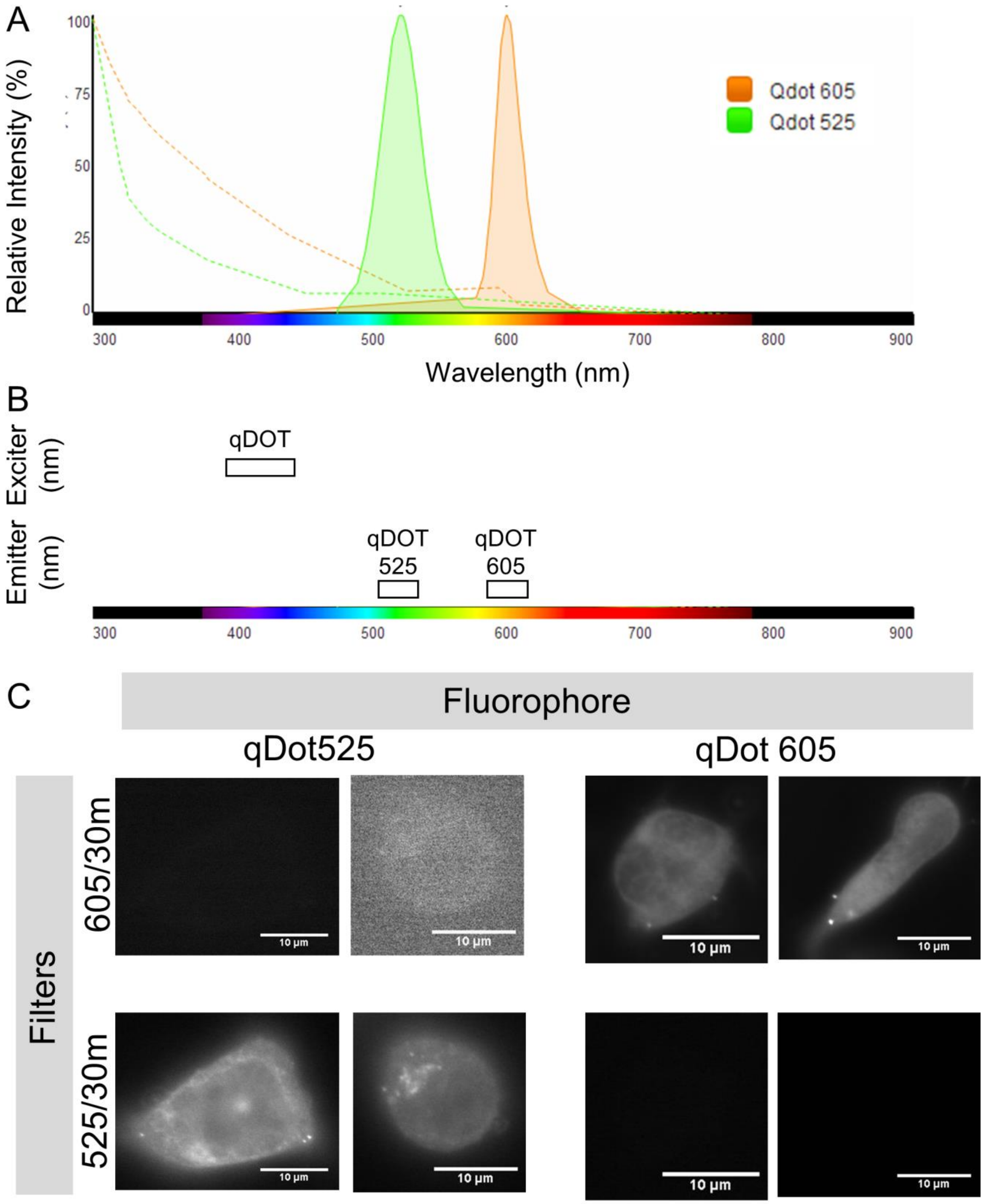

Supplementary Figure 15. Distinction of qDot 525 and qDot 605 signals. The emission wave lengths (solid lines) of qDot 525 and qDot 605 were separated (A), coupled with emission filters of non-overlapping ranges (Emitter lane, B) (images drawn with Fluorescence SpectraViewer, Life Technologies). (C) RNA-FISH signals of qDot 525 and qDot 605 acquired with corresponding and exchanged emission filters. The smRNA-FISH of the *Scl2a3* (qDot 525) and *Malat1* (qDot 605) were carried out as described in Figure 5.

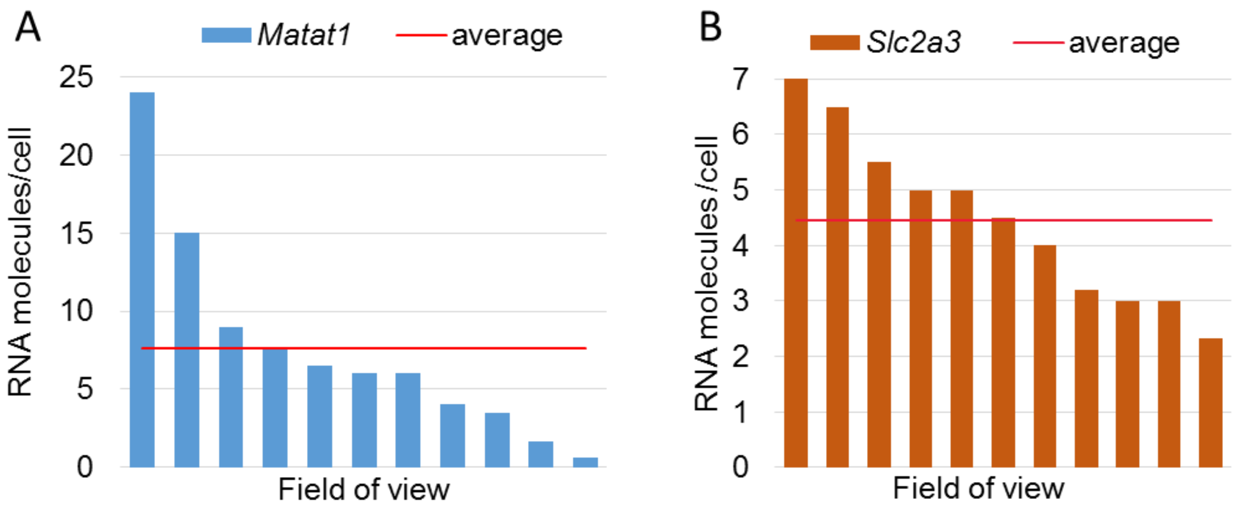

Supplementary Figure 16. Number of RNA molecules per cell identifyed by smRNA-FISH. (A) *Malat1*, (B) *Slc2a3*.

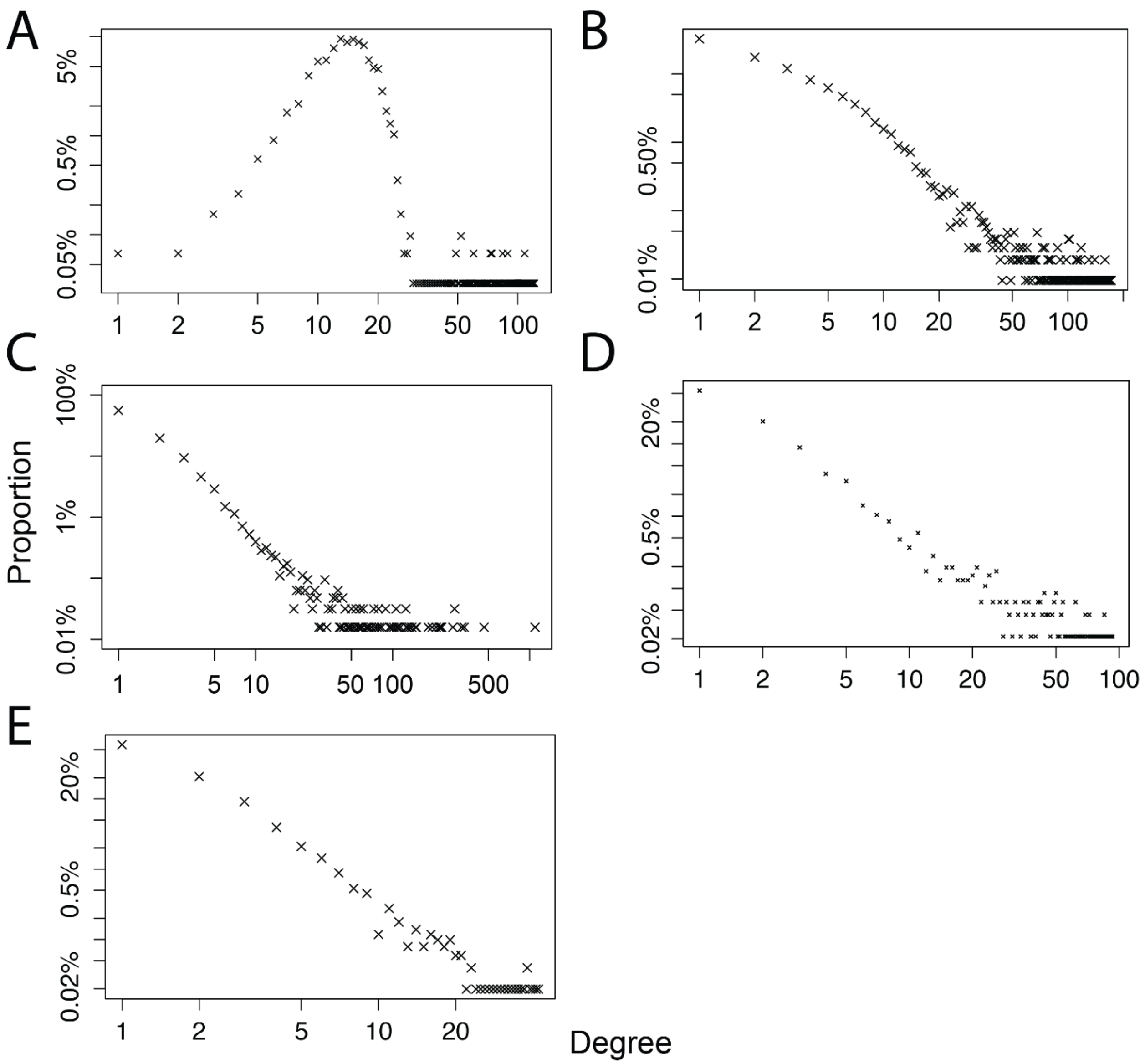

Supplementary Figure 17. Log-log plots for degree distributions of a simulated (A) and experimentally derived RNA-RNA interactomes (B-E). The number of interactions (degrees, x axis) is plotted against the number of notes with this degree (y-axis). (A) An artificial network that contains 100 miRNAs and 1,000 mRNAs. Each miRNA is connected to *N* mRNAs, where *N* is a random number drawn from a uniform distribution between 300 and 1,000. This log-log plot does not exhibit a linear form, as shown in experimentally derived RNA interactomes. (B-C) Experimentally derived RNA-RNA interaction networks of mouse ES cells (B) and brain (C). The number of nodes (RNA) is inversely proportional to their degrees (number of interactions) in the log scale, characteristic of scale-free networks. (D) Same as (B) except that only the interactions supported by 3 or more MARIO reads were included. (E) Same as (B) except that only mRNA, antisense RNA, lincRNA, miRNA, and pseudogene RNA were included.

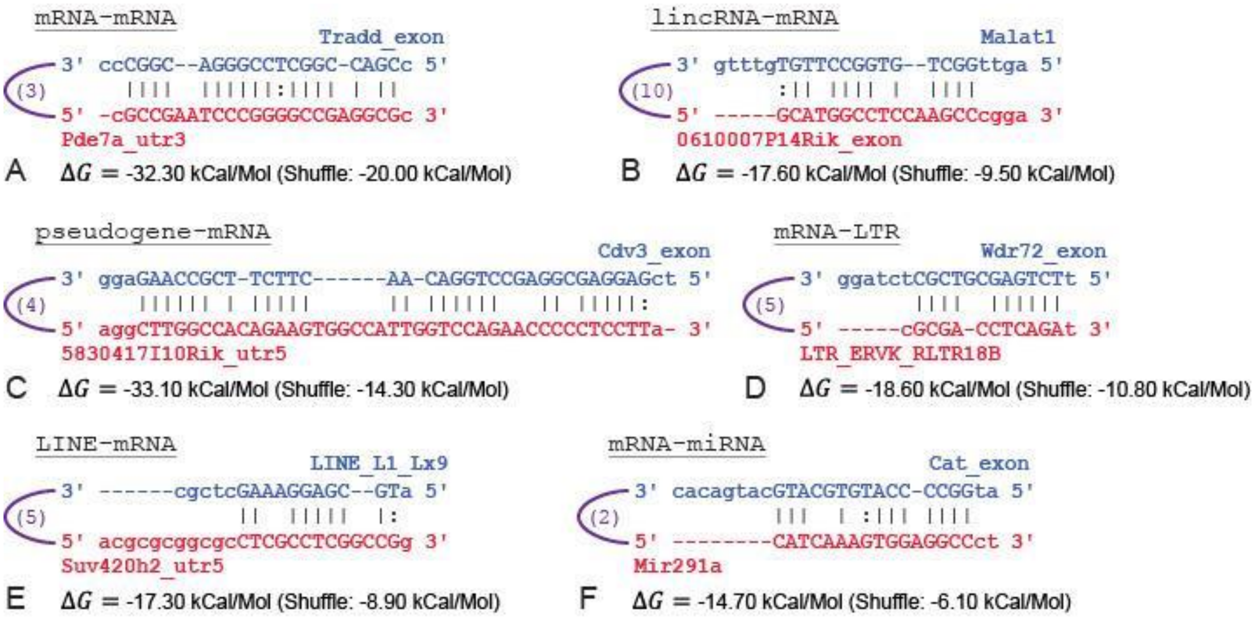

Supplementary Figure 18. Examples of base complementation between MARIO identified interacting RNAs. The types of interacting RNAs included mRNA-mRNA (A), lincRNA-mRNA (B), pseudogeneRNA-mRNA (C), mRNA-LTR (D), LINE-mRNA (E), mRNA-miRNA (F). LTR and LINE represent transposon transcripts. Purple curves represent linker positions. The number of ligated chimeric RNAs supporting each interaction are given in purple brackets. ΔG: hybridization energy. Shuffle: the average hybridization energy of randomly shuffled bases.

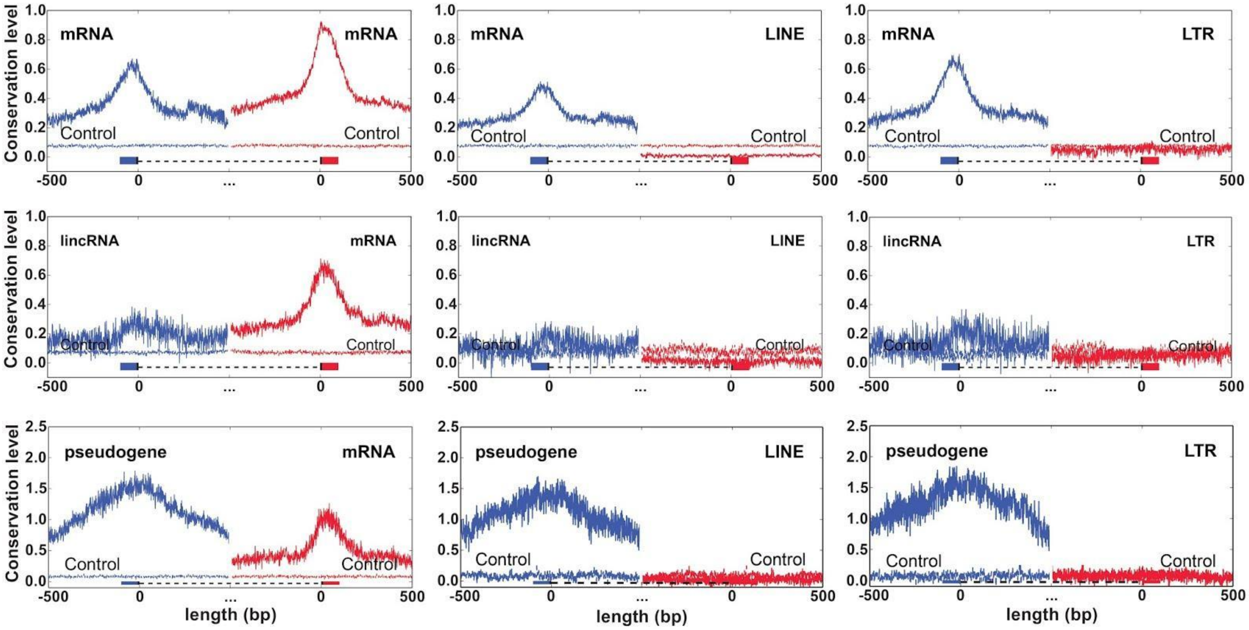

Supplementary Figure 19. Conservation levels of interacting RNAs. Interactions were categorized by RNA types. For each type of interactions, the conservation level was approximated by the average PhyloP scores of the genomic regions (1000bp) centered at the RNA ligation junctions (position 0 on the x axis). The conservation levels of random genomic regions of the same lengths were plotted as controls. Blue and red bars: the RNA1 and RNA2 fragments of a RNA1-Linker-RNA2 chimeric RNA. Dashed line: the linker.

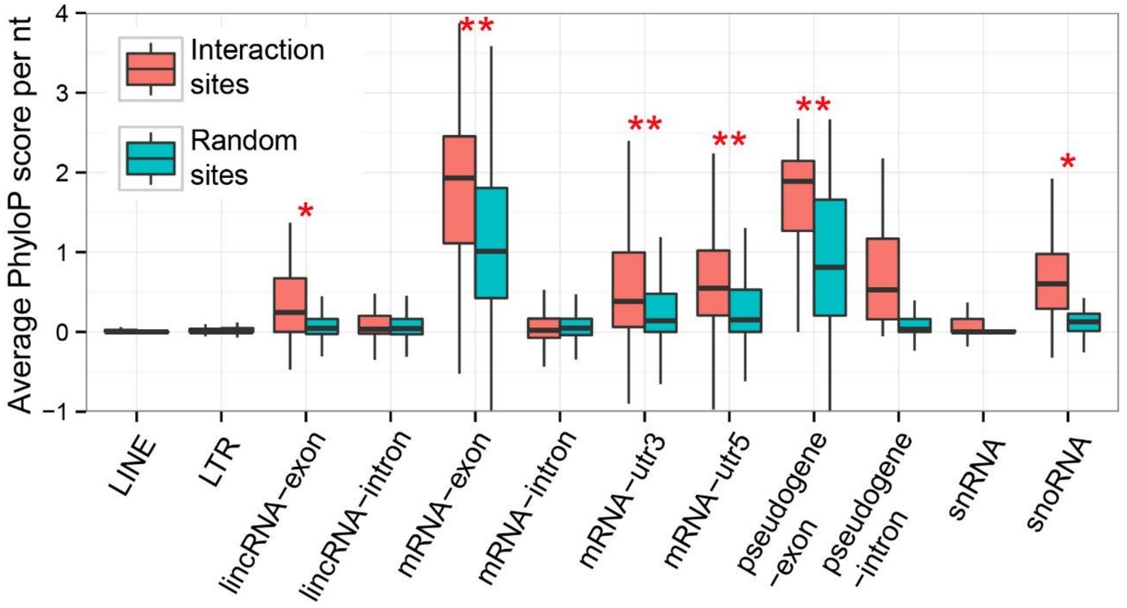

Supplementary Figure 20. Comparison of the conservation levels. Conservation levels were quantified by the average PhyloP score per nucleotide of the interaction sites (y axis). To adjust for the difference of conservation of exons, introns, and UTRs, the interaction sites (red) in annotated exons, introns, and UTRs (dubbed genomic features) were compared to 200,000 randomly sampled genomic sequences from the same genomic feature (blue). The sizes of the randomly sampled genomic sequences shared the same mean and variation as the sizes of interaction sites. P-values were calculated from one-sided two-sample t-test. **: p-value $<10^{-12}$; *: p-value $< 10^{-6}$.

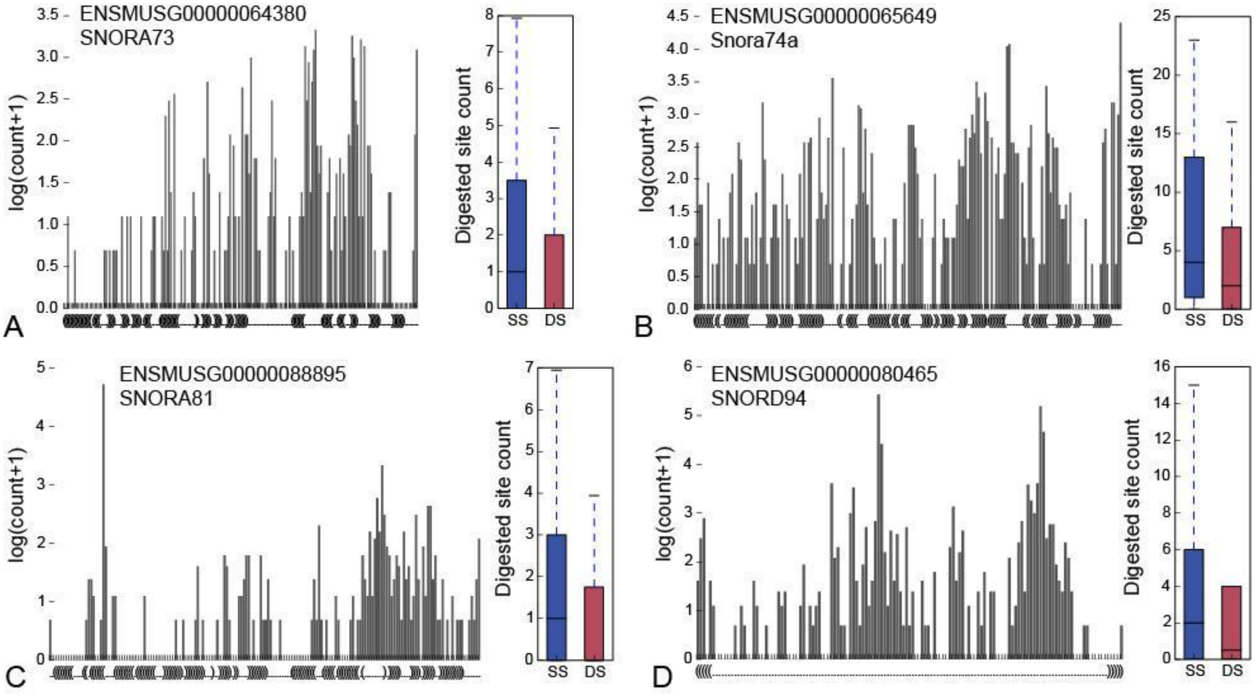

Supplementary Figure 21. Correlation of RNase I digestion density and single-stranded regions. The frequency of digestion measured by the number of read fragments ending or starting at each position (y axis) was compared to known secondary structure (fRNAdb database v3.4) (x axis). Brackets on the x axis represent double-stranded regions. The total counts of read fragments ending or starting at each position in single-stranded (ss) and double-stranded (ds) are summarized on the right panels.

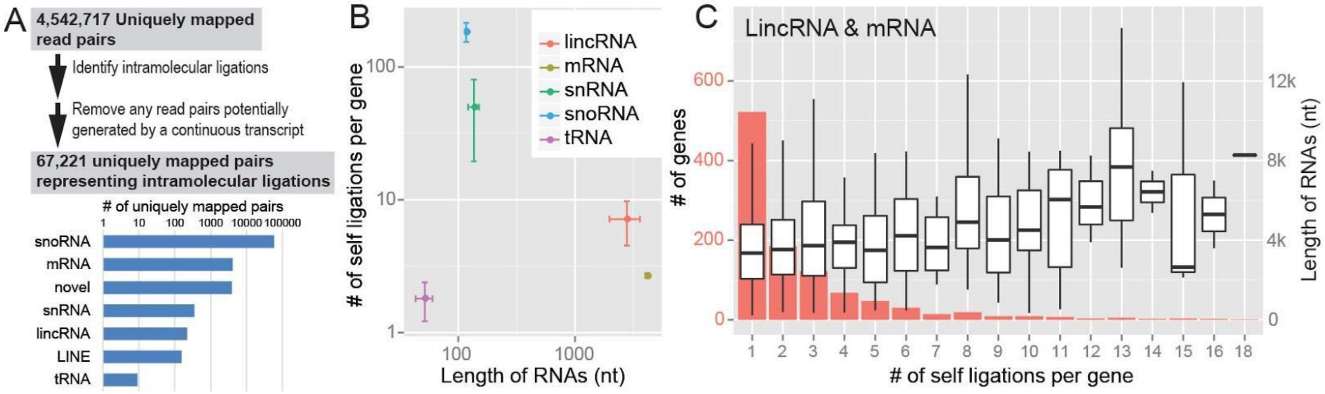

Supplementary Figure 22. Intramolecular ligations. (A) An intramolecular (self) ligation was generated by RNase I digestions of a transcript followed by a linker ligation and a proximity ligation. Therefore, the two RNA fragments on the two sides of the linker came from the same RNA molecule. These intramolecular ligation events were identified with stringent bioinformatic criteria, filtering out pair-end reads that could have been generated from a consecutive transcript. The pair-end reads that could only been generated by a cut-and-ligation process were used for RNA structure analysis. Lower panel: the distribution of intramolecular ligations among different RNA types. (B) The number of intramolecular ligations (y axis) versus the transcript length (x axis) by RNA types. Error bars: standard deviation of the mean. (C) The number (red bars) and the lengths (box plots) of lincRNA and mRNA genes categorized by the number of detected intramolecular ligations (x axis).

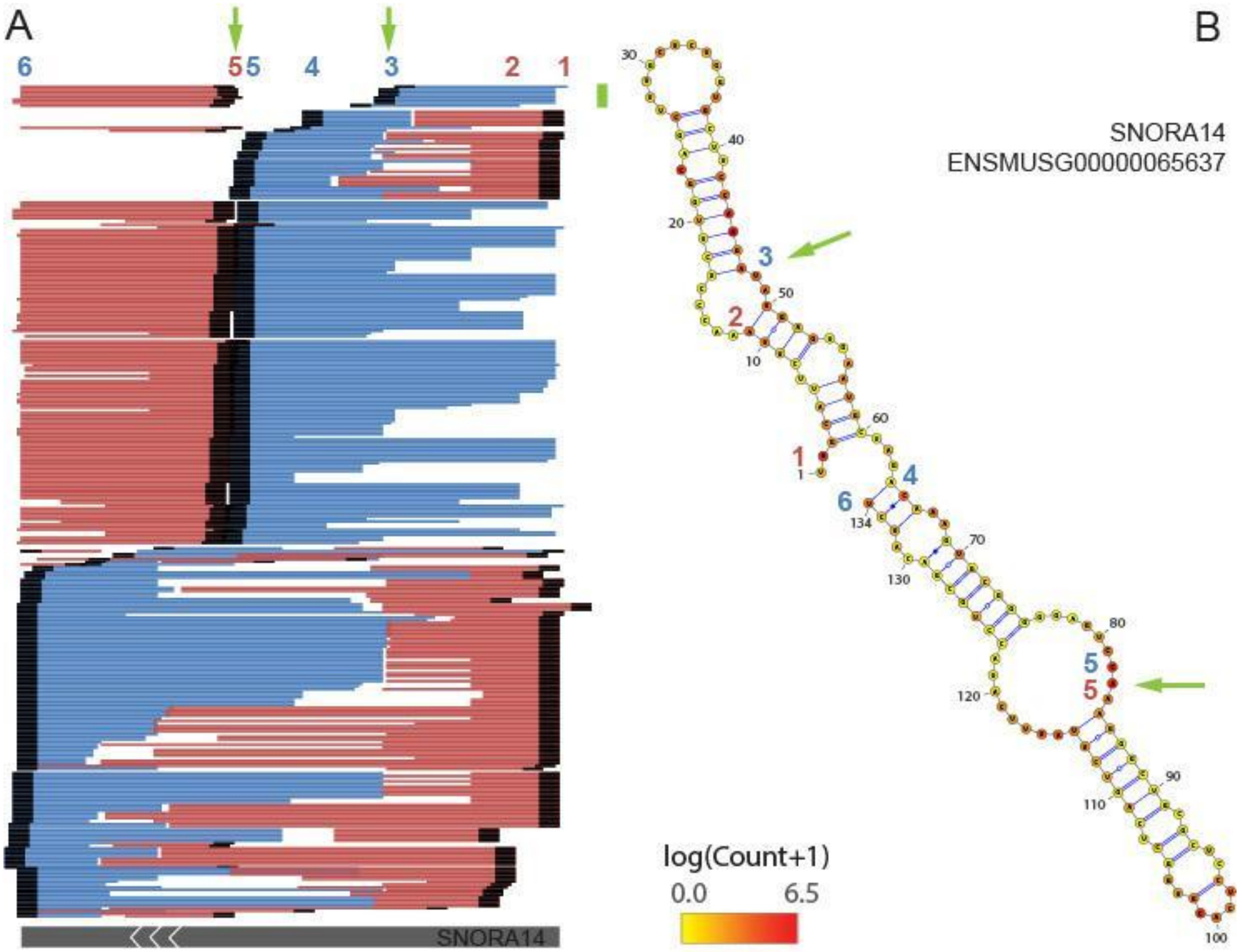

Supplementary Figure 23. MARIO reads on SNORA14. (A) The intramolecular ligation products mapped to *SNORA14*. Black regions: ligation junctions. Colored numbers: positions of dominantly represented ligation junctions at the 5’ (blue numbers) and the 3’ (red) of the linker. Spatial proximities of 1-6, 1-4, and 5(red)-5(blue) positions are consistent with the sequence predicted secondary structure (B). Green arrows point to 3-5 positions which are not close to each other on the sequence predicted secondary structure.

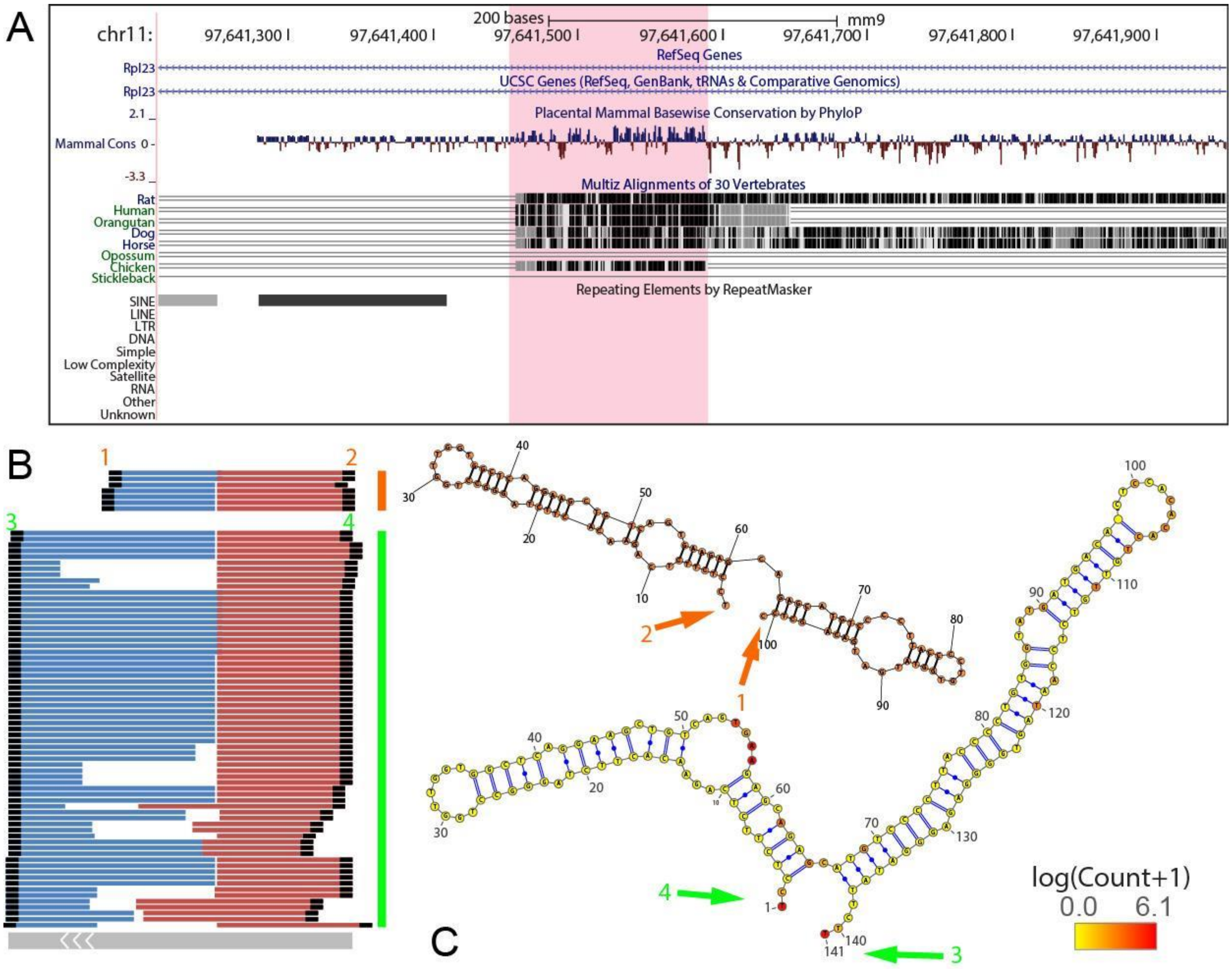

Supplementary Figure 24. A putative novel gene that produces structurally stable transcripts. (A) The genomic location and interspecies conservation of the MARIO predicted novel gene (pink region). (B) The intramolecular ligation products mapped to this novel gene. Black regions: ligation junctions. Colored numbers: positions of dominantly represented ligation junctions. (C) Sequence predicted secondary structures of a long (bottom) and a short (top) transcript produced from this putative gene. The frequency of RNase I digestion on each base (heat map) correlated with the predicted single-stranded regions (bottom). The ligated positions (arrows) are close on the sequenced predicted secondary structures.

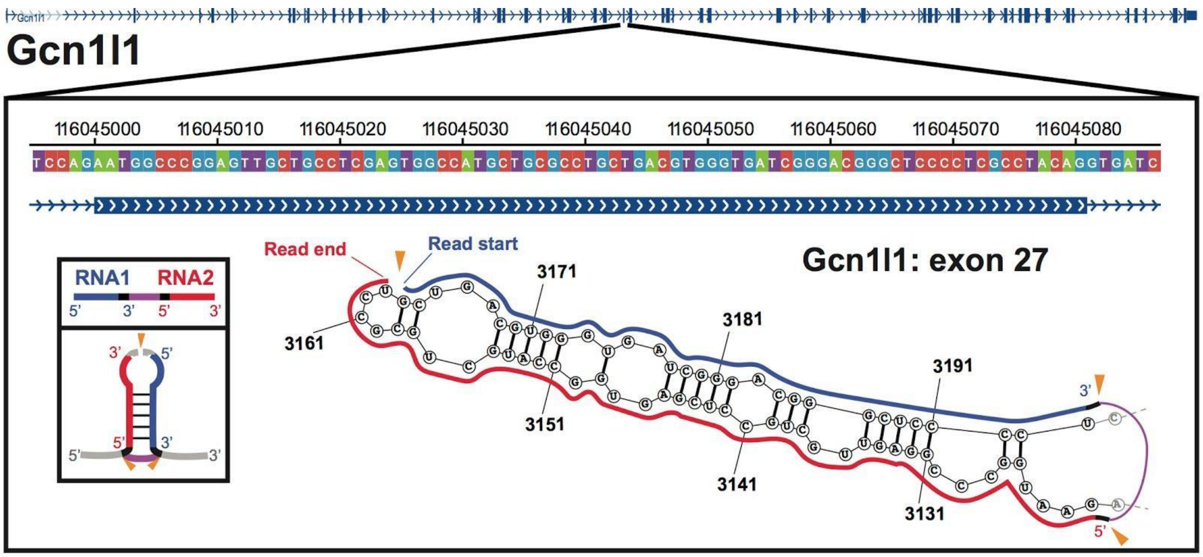

Supplementary Figure 25. The inferred structure of a fraction of an mRNA. An MARIO read pair was superimposed on the secondary structure that was predicted from the sequence of the 27th exon of the Gcn1l1 gene. Blue and red curves correspond to the RNA1 and RNA2 parts of the sequenced chimeric RNA respectively. Purple curve: linker. Black regions on the blue and red curves: ligation junctions. Orange arrows: RNase I cutting positions. The cutting-and-ligation process swapped the 5’-3’ order of two RNA fragments: The 5’ fragment (bases 3122 - 3163, red) and the 3’ fragment (bases 3164 - 3194, blue) of the mRNA were swapped on the sequenced chimeric cDNA (insert).

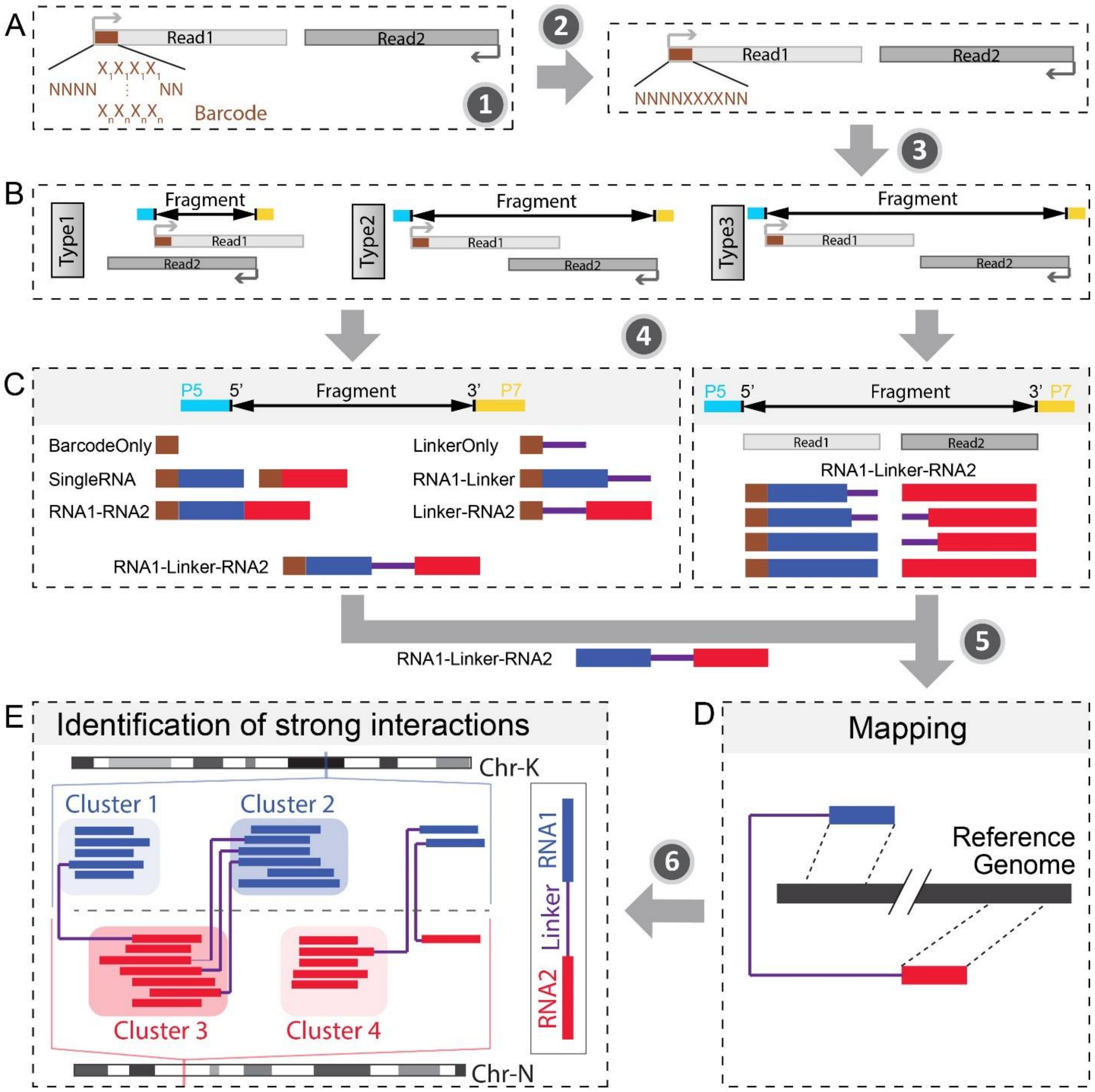

Supplementary Figure 26. The computational pipeline for analysis of MARIO data. (A) PCR duplicates were removed from the pair-end sequencing reads (Step 1). Multiplexed samples were separated based on the 4nt experimental barcodes ('XXXX', Step 2). 'N': a nucleotide of the random barcode. 'X': a nucleotide of the experimental barcode. (B) Each pair of forward (Read1) and reverse (Read2) reads were used to recover a cDNA in the input sequencing library, if possible. (C) The recovered cDNA were categorized based on the configuration of the RNA fragments and the linker sequence (Step 4). The RNA1-Linker-RNA2 type of cDNAs were provided as the output. (D) The RNA1 and the RNA2 parts were separately mapped to the genome. The output was the cDNAs where both RNA1 and RNA2 were uniquely mapped to the genome. (E) RNA-RNA interactions were identified based on association tests.

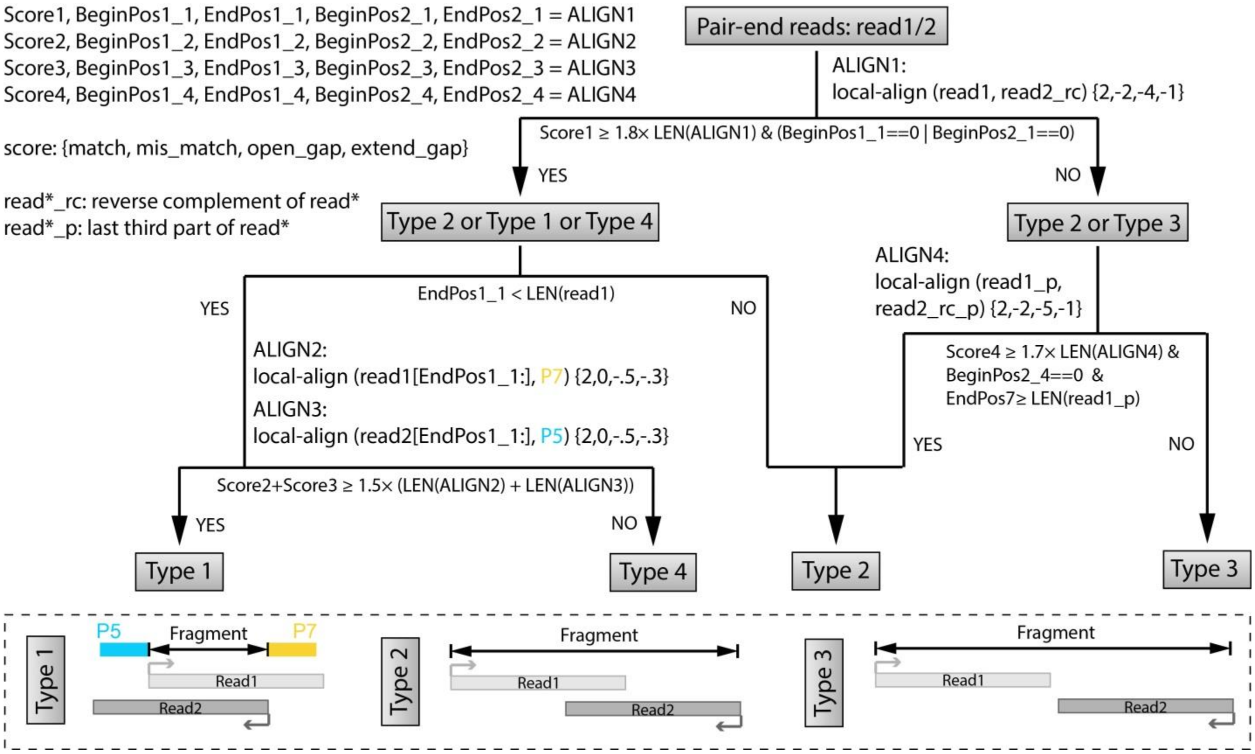

Supplementary Figure 27. The workflow for recovering chimeric cDNAs in the sequencing library. Local alignments were used to identify any overlap between the forward and the reverse reads in a read pair. Local alignments were used four times (ALIGN1 – ALING4) to distinguish four types of possible configurations of any read pair. Three types (Types 1 – 3) were included in the output. Type 1 cDNAs were shorter than 100bp. Type 2 cDNAs were between 100bp and 200bp. Type 3 cDNAs were longer than 200bp. As a quality control, the cDNAs shorter than 100bp but devoid of the known sequence of P5 or P7 sequencing primers were discarded (Type 4). Each alignment is expressed as ‘local-align (seq1,seq2) {M,m,o,e}’, where ‘seq1’ and ‘seq2’ are two input sequences, ‘M’, ‘m’, ‘o’, ‘e’ are parameters for match, mismatch, open-gap and extend-gap penalties. The output of each alignment (X) included the alignment score (ScoreX), the beginning and end positions of the alignment in the first (BeginPos1_X, EndPos1_X) and the second sequence (BeginPos2_X, EndPos2_X).

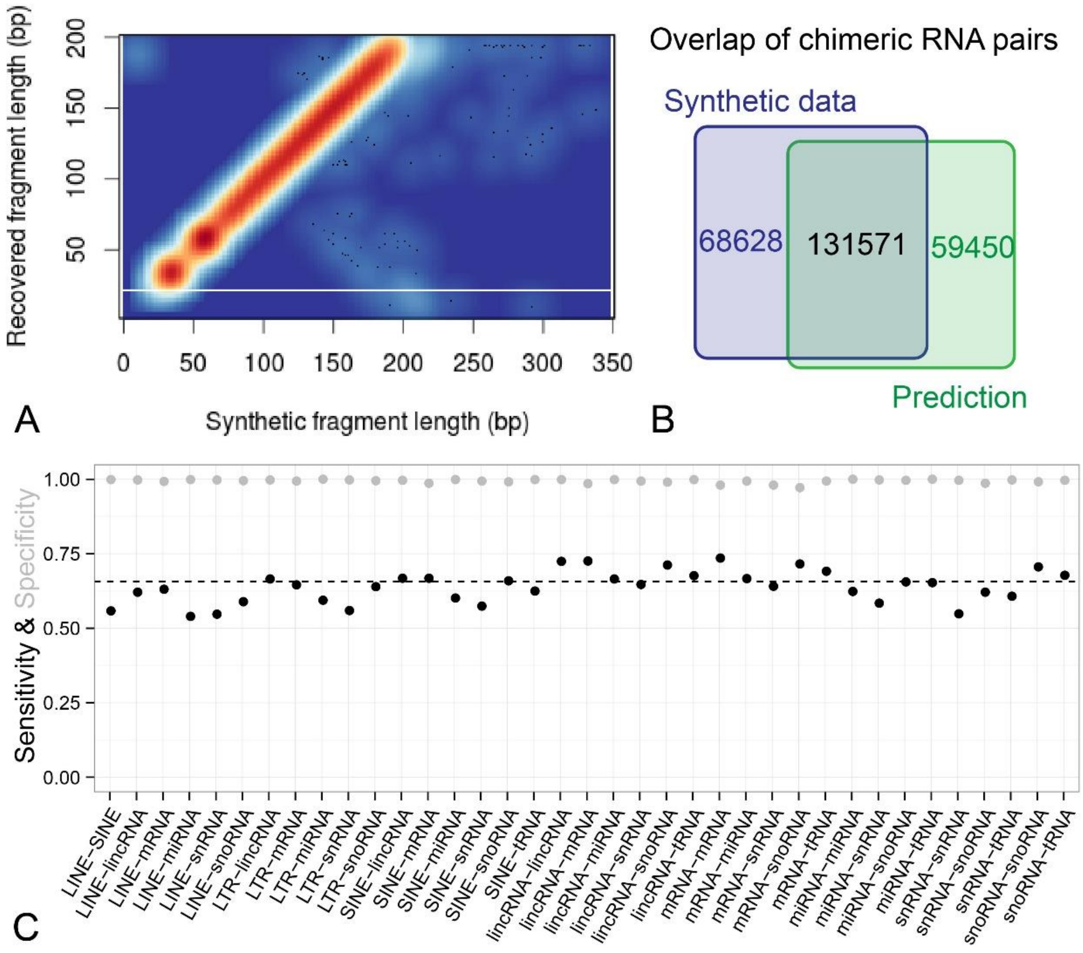

Supplementary Figure 28. Simulation analysis. (A) A scatter plot of the program identified (y axis) and the true lengths of the cDNAs. The cDNAs with program identified lengths greater than 200bp were not included, because their exact lengths could not be calculated. (B) The overlap between the program identified and the simulated RNA pairs. (C) The sensitivity and specificity of the program identified RNA pairs for each type of participating RNAs.

Supplementary tables

**Sample name**	**ES-1**	**ES-2**	**ES-indirect**	**MEF**	**Brain**
Cell type	ES cells	ES cells	ES cells	MEF	Brain Tissue
Crosslinking	254nm UV	254nm UV	Dual crosslinking	254nm UV	254 nm UV
RNA-protein interactions	Direct	Direct	Indirect	Direct	Direct
Protein solubilization	Detergents	Detergents	Sonication	Detergents	Detergents
First fragmentation	1000-2000 nt	~1000 nt	~1000 nt	~300 nt	~1000 nt
rRNA removal	Duplex-specific nuclease	Antibody based	Duplex-specific nuclease	Antibody based	Duplex-specific nuclease
Sample barcode	ACCT	GGCG	AATG	GGCG	TTGT
Total # of read pairs	45,702,794	49,316,127	74,009,386	83,083,324	36,463,565
# of non-duplicate read pairs in the form of RNA1-Linker-RNA2	13,848,413	9,553,722	19,554,316	17,616,980	2,877,233

Supplementary Table 1. Description of the MARIO samples. The “total # of read pairs” is the number of pair-end sequencing reads for each sample. The “# of non-duplicate read pairs in the form of RNA1-Linker-RNA2” is the number of the pair-end reads in the output of Step 4, parsing the chimeric cDNAs, of the bioinformatics pipeline.

Threshold setting	Threshold ES-1	Threshold ES-2	ES-1 unique	Shared	ES-2 unique	Chi-square p value
**1**	3.5E-5	1.4E-5	900	1,651	17,772	< 1E-307
**2**	3.5E-5	2.8E-5	1,288	1,263	3,062	< 1E-307
**3**	3.4E-5	4.2E-5	1,589	1,051	1,272	< 1E-307
**4**	2.4E-6	7.1E-5	27,966	1,146	124	< 1E-307
**5**	4.7E-6	8.5E-5	14,219	949	126	< 1E-307
**6**	1.8E-5	3.6E-4	4,308	247	27	< 1E-307

Supplementary Table 2. The number of unique and overlapping interactions detected in ES-1 and ES-2. Each threshold setting is a threshold for ES-1 (defined as: number of read pairs on a gene pair in ES-1 / total number of mapped read pairs of ES-1) and a threshold for ES-2 (number of read pairs on a gene pair in ES-2 / total number of mapped read pairs of ES-2). The rows are arranged in ascending order for the threshold of ES-2. The Chi-square test was performed for testing the independence of ES-1 and ES-2.

Type	Number of interaction sites	Number of genes containing these sites	Total number of genes in the genome	Total copy number of genes in the genome
mRNA	12439	6600	22562	22562
snoRNA	553	511	1561	1561
tRNA	365	57	60	4760
lincRNA	363	243	2054	2054
snRNA	226	13	32	1429
miRNA	27	25	1630	1630
misc_RNA	33	17	114	487
pseudogene	234	131	5306	5306
antisense	34	31	1351	1351
LINE (L1)	726	76	112	884320
LINE (L2)	26	4	4	65481
LTR (ERVK)	346	96	150	245391
LTR (MaLR)	274	60	102	430745
LTR (ERV1)	235	39	113	61660
LTR (ERVL)	78	31	88	111531
SINE	458	32	40	1521108
Novel	4426			

Supplementary Table 3. Distribution of interaction sites in different types of genes and transposons. Novel: unannotated genomic regions.

Exciter		Emitter		Dye
Wavelength	Bandwidth	Wavelength	Bandwidth	
545	25	605	70	Alexa 555
425	50	525	30	qDot 525
425	50	565	30	qDot 565
425	50	605	30	qDot 605

Supplementary Table 4. Specification of cubes used for imaging.

Identified / True	Type 1	Type 2	Type 3	Type 4	Sensitivity	Specificity
Type 1	**312,411**	24	--	---	99.99%	99.97%
Type 2	65	**480,835**	5,750	898	98.62%	99.73%
Type 3	126	1,322	**197,716**	853	98.84%	99.28%

Supplementary Table 5. A comparison of the program-identified and true cDNA length ranges. The counts of program identified cDNAs of each type (Columns 1 - 4) are compared to their true types (rows).

Identified / True	NoLinker	LinkerOnly	R1-linker	Linker-R2	R1-linker-R2
NoLinker	**266,554**	10	--	--	33,402
LinkerOnly	--	**100,230**	--	--	--
R1-linker	24	25	**100,267**	--	--
Linker-R2	50	58	--	**299,180**	--
R1-linker-R2	57	116	24	22	**199,981**

Supplementary Table 6. A comparison of the program identified and true cDNA configurations. The counts of cDNAs of the program identified configurations (columns) are compared to their true configurations (rows).

Source RNA	# of interaction sites on source RNAs	# of interaction sites on source RNAs with base pairing to target mRNAs	# of interactions with base pairing	Interaction sites on the source RNAs (mm9)
**snoRNA**	172	83	226	http://systemsbio.ucsd.edu/MARIO/Data/OtherRNAs_as_miRNA.htm
**snRNA**	22	8	16	http://systemsbio.ucsd.edu/MARIO/Data/OtherRNAs_as_miRNA.htm
**mRNA**	68	8	8	chr18:48207763-48207972 chr17:13184946-13185035 chr6:67233894-67234046 chr9:64039312-64039420 chr11:69730265-69730433 chr17:6121531-6121797 chr13:45011825-45011869 chr6:115757003-115757184
**LINE**	7	1	8	chr2:90235277-90235370
**Misc_RNA**	4	2	4	chr2:6997218-6997460, chr4:43505643-43505934
**SINE**	3	1	2	chr6:128748868-128748976<br>chr13: 107911768-107911832
**Pseudogene**	13	1	1	chr11:86444105-86444271
**LTR**	5	1	1	chr18:10052120-10052158

Supplementary Table 7. miRNA-like RNAs. The MARIO identified RNA-RNA interactions were filtered by (1) involving an mRNA (dubbed target) and one other RNA (dubbed source RNA), (2) the source RNA was present in smallRNA-seq, (3) both the target and the source RNAs appeared in AGO HITS-CLIP, (4) the MARIO identified interaction sites on the source and the target RNAs exhibit strong base pairing. Column 2 lists the number of interaction sites that satisfied the criteria 1 – 3. Column 3 lists the number of interaction sites that satisfied criteria 1 – 4. Column 4 lists the number of interactions that satisfied criteria 1 – 4.

	Ψ-sites	None Ψ-sites	Total # of ‘U’s	Odds ratio = 4.4
Within RNA interaction sites as detected by MARIO	93	551,541	551,634	
Others	293	7,642,204	7,642,497	P value = 7.70×10^{-95}
Total # of ‘U’s	386	8,193,745	8,194,131	

	Ψ-sites	None Ψ-sites	Total # of ‘U’s	Odds ratio = 10.2
Within snoRNA participated interaction sites as detected by MARIO	57	136,478	136,535	
Others	329	8,057,267	8,057,596	P value < 10^{-100}
Total # of ‘U’s	386	8,193,745	8,194,131	

Supplementary Table 8. Two-way contingency tables for association test of Ψ sites and RNA interaction sites.

Supplementary Notes

Supplementary Note 1. Control experiments for MARIO.

The first control experiment skipped the cross-linking step in the procedure. The second control experiment skipped the protein biotinylation step. The third control experiment carried out the entire procedure on the mixed cell lysate of mouse ES cells and Drosophila S2 cells.

First, we carried out a non-cross-linking control with approximately 3×10^8 mouse ES cells. The RNAs immobilized with proteins on streptavidin beads were purified by protein digestion as previously described. The purified RNAs were subjected to quantification by Qubit RNA HS assay (Invitrogen). The RNAs were below the detection limit of the assay (250 pg/μl). Our sample volume was 20 μl (the same as previously described), which suggests that the RNA abundance was no more than 5 ng. At this point, we stopped the experiment because there was no chance to accomplish linker selection and library construction. In our previously described experiments, the purified RNAs would be in the μg range at this step.

Second, we did another control by not doing protein biotinylation (keeping cross-linking) with 3×10^8 mouse ES cells. It turned out the RNAs purified from the beads were below the detection limit of Qubit RNA HS assay.

Third, we started with 3×10^8 Drosophila S2 cells and 3×10^8 mouse ES cells (cross-species control). The cells were cross-linked and lysed. The lysate from the two cell lines were mixed before protein biotinylation and proximity ligation. The mixture was subjected to the rest of the experimental procedure to produce a sequencing library (Fly-Mm). Fly-Mm contained 27,748,688 read pairs. After removing duplicate reads, 17,330,193 read pairs remained. After splitting by the linker sequence, 3,550,225 read pairs remained, which had the RNA1-RNA2 configuration. Among them, 86,826 (2.5% of 3,550,225) had the two RNA parts mapped to different genomes (RNA1 mapped to dm6 and RNA2 to mm9 and vice versa). Thus, 2.5% of the ligation products were estimated to be generated from random ligations. Furthermore, we asked if this estimate would be affected by assembling the two genomes into a pan-genome (dm9 and mm9) before mapping. A total of 2,697,115 read pairs in the RNA1-RNA2 configuration could be unambiguously mapped to the pan-genome [1,2], among which 184,380 (6.8%) had one RNA part uniquely mapped to the dm9 fraction and the other RNA part uniquely mapped to the mm9 fraction. We chose the more conservative estimate (from the pan-genome method), that 6.8% of the ligation products were generated by random ligations.

Supplementary Note 2. Simulation analysis of MARIO.

1.1 Data synthesis. In order to estimate the sensitivity and specificity of MARIO, including its experimental and computational procedures, we carried out a simulation analysis. We simulated 1,000,000 pair-end reads by computationally mimicking the data generation process. The parameters used for the simulation were derived from real data. The simulated data generation process is as follows.

For each pair-end read (2×100 bases), we:

1. Choose a sample barcode from the four sample barcodes with equal probabilities and concatenate it with a 6nt random barcode (as in Supplementary Figure 26A).
2. Assign this pair-end read to a type of cDNAs from the list of [linkerOnly, NoLinker, RNA1-linker, linker-RNA2, RNA1-linker-RNA2] with probability [0.1, 0.3, 0.1, 0.3, 0.2], respectively (as in Supplementary Figure 26C).
3. If this read-pair was assigned to a linker-containing type, randomly choose 1 or 2 linkers with equal probability. We note that a small percentage of linker-containing read-pairs contained 2 linkers; the use of equal probability was a conservative choice for estimating worst cases.
4. Generate the sequences for the RNA1 and the RNA2 parts, according to the cDNA type determined in Step 2. For both RNA1 and RNA2,
    a. simulate its length from $l \sim Unif(15,150)$,
    b. choose an RNA type from ["miRNA", "mRNA", "lincRNA", "snoRNA", "snRNA", "tRNA"] based on the following probabilities:
        i. if length l <50, use [0.2,0.2,0.1,0.2,0.2,0.1],
        ii. otherwise, use [0.05,0.4,0.2,0.2,0.1,0.05];
    c. randomly choose an RNA according to the sampled RNA type from Ensembl (release 67, mouse NCBIM37),
    d. randomly take a sequence segment with length l from the chosen RNA.
5. Concatenate the barcodes, linker, and RNA fragments generated from Steps 1, 3, 4, producing a synthetic cDNA sequence.
6. If the synthetic cDNA in Step 5 is 100bp or longer, take the 100 bases from the two ends of the synthetic cDNA in forward and reverse strands respectively.
7. If the synthetic cDNA in Step 5 is shorter than 100bp, assign its forward and reverse strands as the forward and the reverse reads, and concatenate P5 and P7 primer sequences to the two reads.
8. Simulate sequencing errors with a rate of 0.01 on each base [3].

Steps 1 – 5 simulated a cDNA sequence according the experimental procedure, and steps 6 – 8 simulated a pair-end read based on this cDNA sequence. The simulated interacting RNA pairs, as well as the cDNA type and the length of each part (RNA1, linker, and RNA2, if applicable) were kept for comparison with the computational predictions.

1.2. Evaluation of intermediate and final results.

We used the synthetic data to evaluate the sensitivities and specificities of two intermediate analysis steps, as well as the final predictions.

First, we compared the program-identified cDNA lengths (output of Step 3 of MARIO-Tools) to the actual (synthesized) lengths (Supplementary Table 5). This step "3. Recovering the cDNAs in the sequencing library" assigns each cDNA into four types with respect to their lengths, namely Type 1 (<100 bp); Type 2 (100~200 bp); Type 3 (>200 bp); Type 4 (unknown) (Supplementary Figure 27). The algorithm achieved high sensitivity and specificity for identifying each type. Only very few (0.58%) of the cDNAs shorter than 200bp were identified as longer than 200bp. These errors were due to a small overlap (typically between 0 and 5 bps) of the forward and the reverse reads, which were not detected by the local alignment.

When the program identified length was shorter than 200 bp (Types 1 and 2), the exact length could be computed. In these cases, the program identified lengths often precisely matched the lengths of the simulated cDNAs (Supplementary Figure 28A).

Next, we compared the program identified chimeric configuration of each cDNA (output of Step 4 of RNA-HiC-Tools) with the synthesized configuration. In Step "4. Parsing the chimeric cDNAs", the algorithm assigned the cDNAs into five categories, based on the presence of the linker sequence. The algorithm reached 99.89% sensitivity and 95.82% specificity for the cDNAs in the "RNA1-linker-RNA2" form (Supplementary Table 6).

Lastly, we compared the program identified and the simulated RNA-RNA interactions. The simulated dataset contained 200,200 chimeric RNA pairs, among which 131,571 pairs of RNAs were detected (sensitivity = 65.72%, specificity = 92.57%, Supplementary Figure 28C). We also separately calculated the sensitivity and specificity for interactions of each type of RNAs (Supplementary Figure 28C). Regardless of the types of participating RNAs, the method showed few false positives (specificity ≥ 90%). Interactions that did not involve transposon RNA or snRNA exhibited fewer false negatives than those that did. This was due to the repetitive nature of transposon and snRNA sequences. The worst cases involved LINE RNAs, where sensitivities dropped to 52%. We therefore conservatively estimate that about a half of the interactions involving transposon RNAs could have been missed by this procedure. We estimate that about 2/3 to 3/4 of the interactions that do not involve transposon RNAs would have been identified.

Supplementary Note 3. Other RNAs with miRNA-like interactions.

We wished to know whether other RNAs could experience a similar process to miRNA biogenesis and also interact with mRNAs. To do so, we intersected the MARIO identified interacting RNAs with those found by small RNA sequencing (smallRNA-seq) and those bond to the AGO protein (HITS-CLIP) in ES cells [4]. The smallRNA-seq selectively sequenced, “miRNAs and other small RNAs that have a 3' hydroxyl group resulting from enzymatic cleavage by Dicer or other RNA processing enzymes” [5]. Besides miRNA, other RNA types including snoRNA, pseudogeneRNA, mRNA UTRs also contributed to the small RNA pool, and were attached to AGO (Supplementary Figure 9A). Moreover, large portions of MARIO identified interacting RNA pairs co-appeared in AGO HITS-CLIP data (Supplementary Figure 10). This data suggest there are non-miRNAs that are digested by DICER or other RNA processing enzymes and are incorporated into the RISC complex.

To elucidate what types of non-miRNA genes were most likely to undergo miRNA-like biogenesis, we subjected the MARIO identified RNA-RNA interactions to the following filters:

1. the interaction involves one mRNA (dubbed target) and one other RNA (source RNA);
2. the source RNA is processed into small RNA by enzymatic cleavage (FPKM>0 in smallRNA-seq);
3. both the target and the source RNAs appear in AGO HITS-CLIP (FPKM>0 for both RNAs);
4. the MARIO identified interaction sites on the source and the target RNAs exhibit strong base pairing (p-value < 0.05, Wilcoxon signed-rank test comparing the binding energies between the RNA1 and RNA2 sequences of every pair-end read to the binding energies of randomly shuffled nucleotide sequences).

A total of 302 RNA-RNA interactions passed these filters. The majority (79%) of the source RNAs in these interactions were snoRNAs (Supplementary Table 7). We therefore prioritized snoRNAs for functional analysis.

We hypothesized that a large number of snoRNAs were enzymatically processed into miRNA-like short RNAs and interact with mRNAs. This hypothesis was supported by 919 MARIO identified snoRNA-mRNA interactions where both the mRNA and the snoRNA were bound by AGO. Furthermore, AGO bound snoRNAs and their interacting mRNAs exhibited anti-correlated expression changes during guided differentiation of ES cells toward mesendoderm [6] (Supplementary Figure 9B). Additionally, AGO bound snoRNAs and their target mRNAs exhibited stronger base pairing than that without AGO binding (Supplementary Figure 9C). Finally, the small RNAs processed from snoRNAs referentially interacted with the UTR regions of mRNAs. Out of the 497 snoRNAs involved in RNA-RNA interactions, 243 interacted with UTR regions, among which 223 (92%) were detected in smallRNA-seq, suggesting the experience of an enzymatic cut (Supplementary Figure 9D). In comparison, the other 254 snoRNAs interacting with non-UTR regions contained fewer (55%) small RNAs. Besides, two times more UTR-interacting sno-siRNAs were AGO bound than the non-UTR interacting snoRNAs (p-value $< 2.2^{-16}$, Chi-square test). For example, *Snora14* RNA targeted the 3’ UTR of *Mcl1* mRNA (Supplementary Figure 11A). The interacting site on *Snora14* RNA (110 - 135nt)

precisely overlapped with the enzymatically processed small RNA (light purple lane) as well as the AGO bound region (green lane). The enzymatically processed portion of *Snora14* RNA is located completely on one side of a hairpin loop (blue line, Supplementary Figure 11B), and exhibits a strong binding affinity (-60 kCal/mol) to the target site on *Mcl1* UTR (red line). The expression of the processed *Snora14* RNA was negatively correlated with that of *Mcl1* mRNA (Supplementary Figure 11C). Taken together, this data suggest a large number of small interfering RNAs originated from snoRNA genes, which interact with more than 900 mRNAs in ES cells.

Supplementary Note 4. Validation by RAP-seq.

We carried out a Malat1 RAP-sequencing experiment on mouse ES cells [7,8]. After cross-linking [8], we used five antisense oligonucleotides to pulldown Malat1 and then sequence the other RNAs that were purified together with Malat1. We did Actin RAP-sequencing as the control. Malat1 RNA itself exhibited a 5.81 fold increase in Malat1 RAP-seq than Actin RAP-seq, confirming the validity of the purification. MARIO reported that Malat1 as a “hub” lincRNA which interacted with Tfrc, Slc2a3, Eif4a2, and 0610007P14Rik RNA. These RNAs showed 14.6 (0610007P14Rik), 4.53 (Slc2a3), 3.38 (Eif4a2), and 2.39 (Tfrc) fold increase in Malat1 RAP-seq than Actin RAP-seq (the largest Chi-square test p-value < 0.0003). This suggests a strong overlap of Malat1 targets from MARIO and Malat1 RAP-seq.

For another validation, we did a Tfrc RAP-seq experiment. Tfrc was identified as a Malat1 interacting RNA from MARIO. We asked whether Tfrc pulldown could reversely identify Malat1. The Tfrc RNA itself showed 2.87 fold of increase in Tfrc RAP-seq compared to Actin RAP-seq. In the same dataset, Malat1 RNA showed 3.84 fold increase, comparing Tfrc RAP-seq to Actin RAP-seq (p –value $< 2.2 \times 10^{-16}$, derived from testing the null hypothesis fold change = 1).

We checked whether the other RNAs interacting with Tfrc as identified by MARIO could be validated by Tfrc RAP-seq as well. MARIO data identified a total of five RNAs as interacting with Tfrc. Besides Malat1, the other four were all snoRNAs, namely Snord13, SNORA3, Snord52, SNORA74. Three of these 4 snoRNAs exhibited fold increases (1.4 fold for Snord13, 13.6 fold for SNORA3, 8.7 fold for SNORA74) in Tfrc RNA-seq as compared to Actin RAP-seq, confirming these interactions (Chi-square test p value < 0.00002). In summary, RAP-seq confirmed nearly all MARIO identified interactions. With the two types of experiments (MARIO and RAP-seq), we now nominate a few RNA interactions (mentioned above) as “real” in mouse ES cells.

Supplementary Note 5. Comparison of snoRNA-mRNA interactions with mRNA pseudouridines.

We compared the pseudouridylation sequencing data (Ψ-seq) [9] with our RNA-interaction sites. Schwartz et al. carried out Ψ-seq in yeast and in mouse bone-marrow-derived dendritic cells (BMDDC). We retrieved the BMDDC Ψ-seq data (CMC treated GSM1464234 and control GSM1464235), and called pseudouridines (Ψ-sites) using the bioinformatic procedure described in the paper. Briefly, Ψ-sites were determined as having more than 5 CMC-treated reads next to a 'U' on the correct strand and direction and having a Ψ-fc value greater than 3. This yielded 386 Ψ-sites out of a total of 8,194,131 'U' positions (0.00471% 'U's were Ψ-sites).

Next, we compared these 386 Ψ-sites to MARIO identified RNA interaction sites. We acknowledge that Ψ-seq and MARIO were done in different cell types. Nevertheless, within our RNA interaction sites, 93 were Ψ-sites out of a total of 551,634 'U's (0.0109%). Therefore, RNA interaction sites determined by MARIO were enriched with Ψ-sites (odds ratio = 4.4, Chi-squared test p-value = 7.70×10^{-95}) (Supplementary Table 8).

Furthermore, we asked whether the Ψ-sites were enriched in the snoRNA-mRNA interaction sites detected by MARIO. Within snoRNA participating interaction sites, there were 57 Ψ-sites out of a total of 136,535 'U's (0.0381%). Compared to the entire transcriptome, MARIO detected snoRNA-participated interaction sites were greatly enriched with Ψ-sites (odds ratio = 10.2, Chi-squared test p-value < 1×10^{-100}) (Supplementary Table 8). Although snoRNA was known to contribute to RNA pseudouridination, these data indicate which snoRNAs may be specifically responsible.

Supplementary References

1 Nagano, T. *et al.* Single-cell Hi-C reveals cell-to-cell variability in chromosome structure. *Nature* **502**, 59-64, doi:10.1038/nature12593 (2013).

2 Helwak, A., Kudla, G., Dudnakova, T. & Tollervey, D. Mapping the human miRNA interactome by CLASH reveals frequent noncanonical binding. *Cell* **153**, 654-665, doi:10.1016/j.cell.2013.03.043 (2013).

3 Loman, N. J. *et al.* Performance comparison of benchtop high-throughput sequencing platforms. *Nature biotechnology* **30**, 434-439, doi:10.1038/nbt.2198 (2012).

4 Chi, S. W., Zang, J. B., Mele, A. & Darnell, R. B. Argonaute HITS-CLIP decodes microRNA-mRNA interaction maps. *Nature* **460**, 479-486, doi:10.1038/nature08170 (2009).

5 Illumina. TruSeq(R) Samll RNA Sample Preparation Guide. 1-43 (2014).

6 Yu, P. *et al.* Spatiotemporal clustering of the epigenome reveals rules of dynamic gene regulation. *Genome research* **23**, 352-364, doi:10.1101/gr.144949.112 (2013).

7 Engreitz, J. M. *et al.* RNA-RNA interactions enable specific targeting of noncoding RNAs to nascent Pre-mRNAs and chromatin sites. *Cell* **159**, 188-199, doi:10.1016/j.cell.2014.08.018 (2014).

8 Kretz, M. *et al.* Control of somatic tissue differentiation by the long non-coding RNA TINCR. *Nature* **493**, 231-235, doi:10.1038/nature11661 (2013).

9 Schwartz, S. *et al.* Transcriptome-wide mapping reveals widespread dynamic-regulated pseudouridylation of ncRNA and mRNA. *Cell* **159**, 148-162, doi:10.1016/j.cell.2014.08.028 (2014).
